# Supplementary material for: Association of sleep duration at age 50, 60, and 70 years with risk of multimorbidity in the UK: 25-year follow-up of the Whitehall II cohort study
Source: PLoS Med. 2022 Oct 18;19(10):e1004109. doi: 10.1371/journal.pmed.1004109 (PMC9578599; doi:10.1371/journal.pmed.1004109)
Supplement: S1 Text — (DOCX) [file pmed.1004109.s002.docx]

**S1 Text. Analysis plan drafted in August 2021 before data analysis**

Study population: Participants with data on sleep measures at the target age (50, 60, or 70) or period (after first disease) and free of multimorbidity at the measure of sleep.

Exposure

1. Sleep duration at age 50, 60, and 70
2. Sleep duration after 1^st^ chronic condition

Outcome

1. Mulitmorbidity defined as at least 2 of the following: cancer, diabetes, CHD, heart failure, COPD, CKD, stroke, depression, dementia, other mental disorders, Parkinson’s disease, liver disease, arthritis/rheumatoid arthritis (cardiovascular risk factors including hypertension not considered as in previous papers; Sindi et al, BMC med, 2020; Vetrano, Plos med, 2018)
2. Transitions in health states: from Healthy state, first chronic condition, multimorbidity, and death for sleep measured at age 50 among those free of a first chronic disease at sleep measurement (age 50 chosen as disease less prevalent at that age to reduce selection bias)
3. Transitions in health states: from first chronic disease, multimorbidity, and death for sleep duration after first chronic disease among those with a first chronic disease during the follow-up

Covariates

1. Model 1: Age (exact age, time-scale), sex, ethnicity, education (continuous), grade (3 classes, categorical), marital status
2. Model 2: Model 1 + smoking, alcohol, fruits & vegetables, physical activity (number of hours of MVPA, continuous), BMI (4 classes), hypertension, sleep medication

Prevalence of one chronic disease was added to the health-related adjustment variables (Model 3) in analysis on the association between sleep duration at different ages and risk of multimorbidity at the revision stage.

Analyses

1. Analysis 1: Sleep duration at age 50, 60, 70 and multimorbidity using Cox regressions
2. Analysis 2: Sleep duration at age 50 and the course of chronic disease using multi-state model
3. Analysis 3: sleep duration after a first chronic condition and incidence of multimorbidity and death using a multi-state model among those with one chronic condition (consider inverse probability weighting to account for missing data)

Interactions to consider: sex

Sensitivity analysis

1. Repeat analysis for sleep duration at age 50, 60, and 70 and multimorbidity excluding participants with a first chronic disease
2. Repeat analysis for sleep duration at age 50, 60, and 70 and multimorbidity excluding participants using sleep medication
3. Examine the association between accelerometer-assessed sleep duration (assessed in 2012-13) and incident multimorbidity
