# Supplementary figures and images for: Association of sleep duration at age 50, 60, and 70 years with risk of multimorbidity in the UK: 25-year follow-up of the Whitehall II cohort study
Source: PLoS Med. 2022 Oct 18;19(10):e1004109. doi: 10.1371/journal.pmed.1004109 (PMC9578599; doi:10.1371/journal.pmed.1004109)

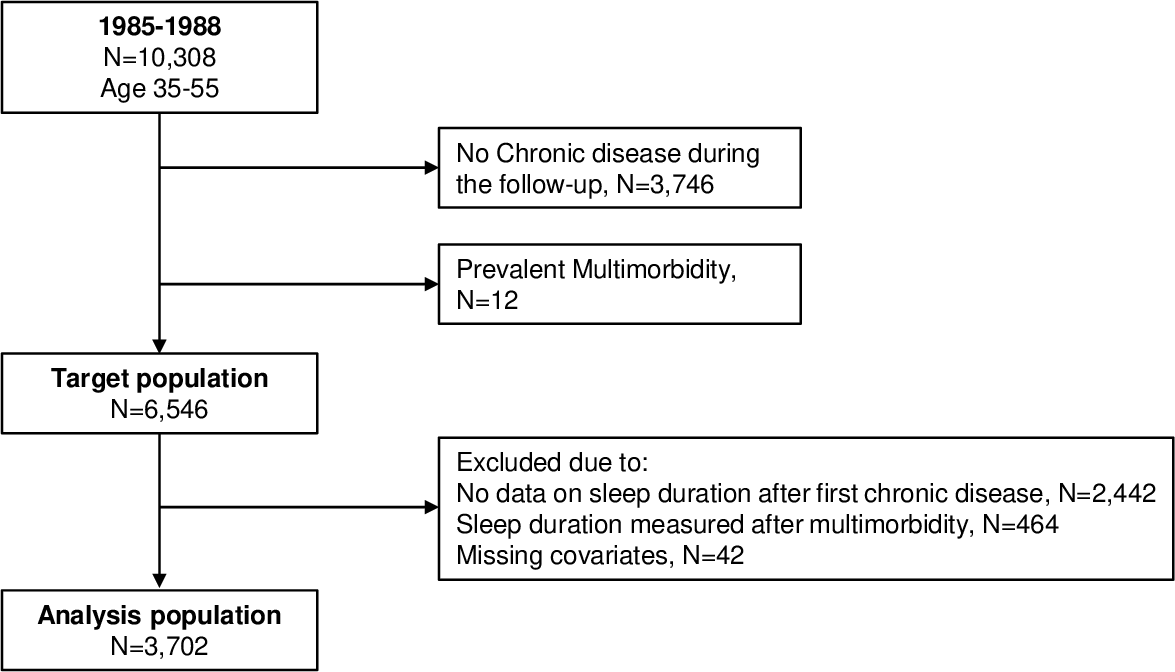

Supplement: S1 Fig — (TIF) [file pmed.1004109.s003.tif]
