# Supplementary material for: Association of sleep duration at age 50, 60, and 70 years with risk of multimorbidity in the UK: 25-year follow-up of the Whitehall II cohort study
Source: PLoS Med. 2022 Oct 18;19(10):e1004109. doi: 10.1371/journal.pmed.1004109 (PMC9578599; doi:10.1371/journal.pmed.1004109)
Supplement: S1 Table — (DOCX) [file pmed.1004109.s004.docx]

**S1 Table. Characteristics of the study population at age 60**

|  |  |  | **Sleep duration at age 60** | | | | |  |
| --- | --- | --- | --- | --- | --- | --- | --- | --- |
|  | **Total** |  | **≤5 hours** | **6 hours** | **7 hours** | **8 hours** | **≥9 hours** | **P** |
| N | 6,848 |  | 519 | 2,095 | 2,882 | 1,230 | 122 |  |
| Sex |  |  |  |  |  |  |  | <0.001 |
| Men | 4,820 (70.4) |  | 314 (60.5) | 1,452 (69.3) | 2,094 (72.7) | 879 (71.5) | 81 (66.4) |  |
| Women | 2,028 (29.6) |  | 205 (39.5) | 643 (30.7) | 788 (27.3) | 351 (28.5) | 41 (33.6) |  |
| Ethnicity |  |  |  |  |  |  |  | <0.001 |
| White | 6,314 (92.2) |  | 447 (86.1) | 1,908 (91.1) | 2,703 (93.8) | 1,152 (93.7) | 104 (85.2) |  |
| Non-white | 534 (7.8) |  | 72 (13.9) | 187 (8.9) | 179 (6.2) | 78 (6.3) | 18 (14.8) |  |
| Education |  |  |  |  |  |  |  | <0.001 |
| Primary school or less | 807 (11.8) |  | 100 (19.3) | 241 (11.5) | 312 (10.8) | 143 (11.6) | 11 (9.0) |  |
| Lower secondary school | 2,197 (32.1) |  | 173 (33.3) | 671 (32.0) | 921 (32.0) | 396 (32.2) | 36 (29.5) |  |
| Higher secondary school | 1,829 (26.7) |  | 129 (24.9) | 562 (26.8) | 777 (27.0) | 325 (26.4) | 36 (29.5) |  |
| University | 1,512 (22.1) |  | 94 (18.1) | 467 (22.3) | 648 (22.5) | 274 (22.3) | 29 (23.8) |  |
| Higher degree | 503 (7.3) |  | 23 (4.4) | 154 (7.4) | 224 (7.8) | 92 (7.5) | 10 (8.2) |  |
| Occupational position |  |  |  |  |  |  |  | <0.001 |
| Low | 882 (12.9) |  | 127 (24.5) | 287 (13.7) | 320 (11.1) | 130 (10.6) | 18 (14.8) |  |
| Intermediate | 2,875 (42.0) |  | 246 (47.4) | 902 (43.1) | 1,169 (40.6) | 508 (41.3) | 50 (41.0) |  |
| High | 3,091 (45.1) |  | 146 (28.1) | 906 (43.3) | 1,393 (48.3) | 592 (48.1) | 54 (44.3) |  |
| Marital status |  |  |  |  |  |  |  | <0.001 |
| Married/cohabiting | 5,235 (76.4) |  | 328 (63.2) | 1,560 (74.5) | 2,269 (78.7) | 987 (80.2) | 91 (74.6) |  |
| Single/divorced/widowed | 1,613 (23.6) |  | 191 (36.8) | 535 (25.5) | 613 (21.3) | 243 (19.8) | 31 (25.4) |  |

**S1 Table (Continued).**

|  |  |  | **Sleep duration at age 60** | | | | |  |
| --- | --- | --- | --- | --- | --- | --- | --- | --- |
|  | **Total** |  | **≤5 hours** | **6 hours** | **7 hours** | **8 hours** | **≥9 hours** | **P** |
| Smoking status |  |  |  |  |  |  |  | 0.767 |
| Never smoker | 3,297 (48.1) |  | 254 (48.9) | 1,012 (48.3) | 1,375 (47.7) | 599 (48.7) | 57 (46.7) |  |
| Ex-smoker | 2,929 (42.8) |  | 209 (40.3) | 884 (42.2) | 1,260 (43.7) | 522 (42.4) | 54 (44.3) |  |
| Current smoker | 622 (9.1) |  | 56 (10.8) | 199 (9.5) | 247 (8.6) | 109 (8.9) | 11 (9.0) |  |
| Alcohol consumption |  |  |  |  |  |  |  | <0.001 |
| 0 unit/week | 1,184 (17.3) |  | 131 (25.2) | 362 (17.3) | 469 (16.3) | 195 (15.9) | 27 (22.1) |  |
| 1-14 units/week | 3,567 (52.1) |  | 264 (50.9) | 1,067 (50.9) | 1,526 (52.9) | 653 (53.1) | 57 (46.7) |  |
| >14 units/week | 2,097 (30.6) |  | 124 (23.9) | 666 (31.8) | 887 (30.8) | 382 (31.1) | 38 (31.1) |  |
| Fruit and vegetable consumption |  |  |  |  |  |  |  | <0.001 |
| Less than once a day | 1,659 (24.2) |  | 158 (30.4) | 533 (25.4) | 672 (23.3) | 265 (21.5) | 31 (25.4) |  |
| Once a day | 2,427 (35.4) |  | 189 (36.4) | 737 (35.2) | 1,027 (35.6) | 426 (34.6) | 48 (39.3) |  |
| Twice or more a day | 2,762 (40.3) |  | 172 (33.1) | 825 (39.4) | 1,183 (41.0) | 539 (43.8) | 43 (35.2) |  |
| Moderate-to-vigorous physical activity (hours), M(SD) | 3.7 (3.5) |  | 3.0 (3.1) | 3.6 (3.6) | 3.8 (3.5) | 4.2 (3.7) | 3.2 (2.9) | <0.001 |
| BMI (kg/m²), M(SD) | 26.4 (4.2) |  | 27.0 (4.7) | 26.7 (4.3) | 26.3 (4.0) | 26.1 (4.1) | 26.6 (4.8) | <0.001 |
| <18.5 kg/m² | 63 (0.9) |  | 8 (1.5) | 19 (0.9) | 26 (0.9) | 9 (0.7) | 1 (0.8) | <0.001 |
| 18.5-24.9 kg/m² | 2,678 (39.1) |  | 179 (34.5) | 766 (36.6) | 1,165 (40.4) | 521 (42.4) | 47 (38.5) |  |
| 25-29.9 kg/m² | 2,942 (43.0) |  | 218 (42.0) | 912 (43.5) | 1,241 (43.1) | 519 (42.2) | 52 (42.6) |  |
| ≥30 kg/m² | 1,165 (17.0) |  | 114 (22.0) | 398 (19.0) | 450 (15.6) | 181 (14.7) | 22 (18.0) |  |
| Hypertension | 2,495 (36.4) |  | 235 (45.3) | 724 (34.6) | 1,048 (36.4) | 446 (36.3) | 42 (34.4) | <0.001 |
| Use of sleep medication | 55 (0.8) |  | 15 (2.9) | 22 (1.1) | 13 (0.5) | 4 (0.3) | 1 (0.8) | <0.001 |
| Prevalence of one chronic disease^a^ at age 60 | 1,421 (20.8) |  | 142 (27.4) | 435 (20.8) | 566 (19.6) | 250 (20.3) | 28 (23.0) | 0.002 |

Abbreviations: BMI, body mass index; M, mean; SD, standard deviation. Values are No. (%) unless stated otherwise.

^a^ Chronic disease among diabetes, cancer, coronary heart disease, stroke, heart failure, chronic obstructive pulmonary disease, chronic kidney disease, liver disease, depression, dementia, other mental disorder, Parkinson’s disease, and arthritis/rheumatoid arthritis.
