# Supplementary material for: Association of sleep duration at age 50, 60, and 70 years with risk of multimorbidity in the UK: 25-year follow-up of the Whitehall II cohort study
Source: PLoS Med. 2022 Oct 18;19(10):e1004109. doi: 10.1371/journal.pmed.1004109 (PMC9578599; doi:10.1371/journal.pmed.1004109)
Supplement: S2 Table — (DOCX) [file pmed.1004109.s005.docx]

**S2 Table. Characteristics of the study population at age 70**

|  |  |  | **Sleep duration at age 70** | | | | |  |
| --- | --- | --- | --- | --- | --- | --- | --- | --- |
|  | **Total** |  | **≤5 hours** | **6 hours** | **7 hours** | **8 hours** | **≥9 hours** | **P** |
| N | 5,546 |  | 451 | 1,574 | 2,249 | 1,151 | 121 |  |
| Sex |  |  |  |  |  |  |  | <0.001 |
| Men | 3,957 (71.3) |  | 263 (58.3) | 1,043 (66.3) | 1,707 (75.9) | 854 (74.2) | 90 (74.4) |  |
| Women | 1,589 (28.7) |  | 188 (41.7) | 531 (33.7) | 542 (24.1) | 297 (25.8) | 31 (25.6) |  |
| Ethnicity |  |  |  |  |  |  |  | <0.001 |
| White | 5,146 (92.8) |  | 400 (88.7) | 1,440 (91.5) | 2,122 (94.4) | 1,077 (93.6) | 107 (88.4) |  |
| Non-white | 400 (7.2) |  | 51 (11.3) | 134 (8.5) | 127 (5.6) | 74 (6.4) | 14 (11.6) |  |
| Education |  |  |  |  |  |  |  | 0.022 |
| Primary school or less | 674 (12.2) |  | 79 (17.5) | 204 (13.0) | 258 (11.5) | 123 (10.7) | 10 (8.3) |  |
| Lower secondary school | 1,853 (33.4) |  | 161 (35.7) | 544 (34.6) | 724 (32.2) | 384 (33.4) | 40 (33.1) |  |
| Higher secondary school | 1,416 (25.5) |  | 100 (22.2) | 395 (25.1) | 580 (25.8) | 309 (26.8) | 32 (26.4) |  |
| University | 1,195 (21.5) |  | 79 (17.5) | 327 (20.8) | 507 (22.5) | 253 (22.0) | 29 (24.0) |  |
| Higher degree | 408 (7.4) |  | 32 (7.1) | 104 (6.6) | 180 (8.0) | 82 (7.1) | 10 (8.3) |  |
| Occupational position |  |  |  |  |  |  |  | <0.001 |
| Low | 610 (11.0) |  | 86 (19.1) | 209 (13.3) | 190 (8.4) | 114 (9.9) | 11 (9.1) |  |
| Intermediate | 2,266 (40.9) |  | 206 (45.7) | 661 (42.0) | 896 (39.8) | 453 (39.4) | 50 (41.3) |  |
| High | 2,670 (48.1) |  | 159 (35.3) | 704 (44.7) | 1,163 (51.7) | 584 (50.7) | 60 (49.6) |  |
| Marital status |  |  |  |  |  |  |  | <0.001 |
| Married/cohabiting | 4,143 (74.7) |  | 264 (58.5) | 1,120 (71.2) | 1,747 (77.7) | 924 (80.3) | 88 (72.7) |  |
| Single/divorced/widowed | 1,403 (25.3) |  | 187 (41.5) | 454 (28.8) | 502 (22.3) | 227 (19.7) | 33 (27.3) |  |

**S2 Table (Continued).**

|  |  |  | **Sleep duration at age 70** | | | | |  |
| --- | --- | --- | --- | --- | --- | --- | --- | --- |
|  | **Total** |  | **≤5 hours** | **6 hours** | **7 hours** | **8 hours** | **≥9 hours** | **P** |
| Smoking status |  |  |  |  |  |  |  | 0.741 |
| Never smoker | 2,576 (46.4) |  | 211 (46.8) | 747 (47.5) | 1,041 (46.3) | 521 (45.3) | 56 (46.3) |  |
| Ex-smoker | 2,685 (48.4) |  | 211 (46.8) | 745 (47.3) | 1,104 (49.1) | 565 (49.1) | 60 (49.6) |  |
| Current smoker | 285 (5.1) |  | 29 (6.4) | 82 (5.2) | 104 (4.6) | 65 (5.6) | 5 (4.1) |  |
| Alcohol consumption |  |  |  |  |  |  |  | <0.001 |
| 0 unit/week | 1,142 (20.6) |  | 133 (29.5) | 340 (21.6) | 404 (18.0) | 242 (21.0) | 23 (19.0) |  |
| 1-14 units/week | 3,055 (55.1) |  | 232 (51.4) | 858 (54.5) | 1,294 (57.5) | 615 (53.4) | 56 (46.3) |  |
| >14 units/week | 1,349 (24.3) |  | 86 (19.1) | 376 (23.9) | 551 (24.5) | 294 (25.5) | 42 (34.7) |  |
| Fruit and vegetable consumption |  |  |  |  |  |  |  | <0.001 |
| Less than once a day | 1,139 (20.5) |  | 124 (27.5) | 371 (23.6) | 400 (17.8) | 217 (18.9) | 27 (22.3) |  |
| Once a day | 1,656 (29.9) |  | 128 (28.4) | 467 (29.7) | 655 (29.1) | 367 (31.9) | 39 (32.2) |  |
| Twice or more a day | 2,751 (49.6) |  | 199 (44.1) | 736 (46.8) | 1,194 (53.1) | 567 (49.3) | 55 (45.5) |  |
| Moderate-to-vigorous physical activity (hours), M(SD) | 3.8 (3.6) |  | 2.8 (2.9) | 3.6 (3.5) | 4.1 (3.5) | 4.1 (4.0) | 3.8 (3.5) | <0.001 |
| BMI (kg/m²), M(SD) | 26.5 (4.3) |  | 27.1 (4.8) | 26.7 (4.4) | 26.4 (4.1) | 26.3 (4.1) | 26.7 (4.6) | 0.001 |
| <18.5 kg/m² | 61 (1.1) |  | 7 (1.6) | 15 (1.0) | 29 (1.3) | 9 (0.8) | 1 (0.8) | 0.007 |
| 18.5-24.9 kg/m² | 2,091 (37.7) |  | 144 (31.9) | 584 (37.1) | 870 (38.7) | 447 (38.8) | 46 (38.0) |  |
| 25-29.9 kg/m² | 2,411 (43.5) |  | 197 (43.7) | 667 (42.4) | 996 (44.3) | 505 (43.9) | 46 (38.0) |  |
| ≥30 kg/m² | 983 (17.7) |  | 103 (22.8) | 308 (19.6) | 354 (15.7) | 190 (16.5) | 28 (23.1) |  |
| Hypertension | 2,302 (41.5) |  | 206 (45.7) | 646 (41.0) | 932 (41.4) | 465 (40.4) | 53 (43.8) | 0.377 |
| Use of sleep medication | 47 (0.8) |  | 13 (2.9) | 18 (1.1) | 12 (0.5) | 4 (0.3) | 0 (0.0) | <0.001 |
| Prevalence of one chronic disease^a^ at age 70 | 1,898 (34.2) |  | 194 (43.0) | 561 (35.6) | 725 (32.2) | 381 (33.1) | 37 (30.6) | <0.001 |

Abbreviations: BMI, body mass index; M, mean; SD, standard deviation. Values are No. (%) unless stated otherwise.

^a^ Chronic disease among diabetes, cancer, coronary heart disease, stroke, heart failure, chronic obstructive pulmonary disease, chronic kidney disease, liver disease, depression, dementia, other mental disorder, Parkinson’s disease, and arthritis/rheumatoid arthritis.
