# Supplementary material for: Association of sleep duration at age 50, 60, and 70 years with risk of multimorbidity in the UK: 25-year follow-up of the Whitehall II cohort study
Source: PLoS Med. 2022 Oct 18;19(10):e1004109. doi: 10.1371/journal.pmed.1004109 (PMC9578599; doi:10.1371/journal.pmed.1004109)
Supplement: S3 Table — (DOCX) [file pmed.1004109.s006.docx]

**S3 Table. Description of chronic disease dyads in the analysis of sleep duration at age 50, 60 and 70 and incident multimorbidity**

| **Chronic disease dyad** | **Study population at age 50** | | |  | **Study population at age 60** | | |  | **Study population at age 70** | | |
| --- | --- | --- | --- | --- | --- | --- | --- | --- | --- | --- | --- |
|  | **N (%)^a^** | **Rank** | **Mean age at onset (SD)** |  | **N (%)^a^** | **Rank** | **Mean age at onset (SD)** |  | **N (%)^a^** | **Rank** | **Mean age at onset (SD)** |
| Coronary heart disease & diabetes | 305 (11.5) | 1 | 67.4 (7.4) |  | 189 (9.3) | 1 | 69.7 (5.9) |  | 103 (7.3) | 4 | 74.2 (4.4) |
| Coronary heart disease & arthritis/rheumatoid arthritis | 198 (7.4) | 2 | 71.8 (6.8) |  | 170 (8.4) | 2 | 72.4 (6.3) |  | 116 (8.3) | 1 | 76.4 (4.7) |
| Coronary heart disease & cancer | 193 (7.3) | 3 | 69.9 (7.1) |  | 165 (8.1) | 3 | 71.2 (6.0) |  | 112 (8.0) | 2 | 75.2 (4.2) |
| Diabetes & cancer | 174 (6.5) | 4 | 69.8 (7.1) |  | 134 (6.6) | 5 | 70.5 (5.9) |  | 82 (5.8) | 5 | 75.1 (4.3) |
| Arthritis/rheumatoid arthritis & cancer | 158 (5.9) | 5 | 72.9 (7.4) |  | 152 (7.5) | 4 | 72.8 (5.5) |  | 110 (7.8) | 3 | 75.6 (4.7) |
| Coronary heart disease & depression | 143 (5.4) | 6 | 66.4 (7.6) |  | 94 (4.6) | 7 | 69.2 (6.3) |  | 51 (3.6) | 8 | 74.6 (4.8) |
| Diabetes & arthritis/rheumatoid arthritis | 134 (5.0) | 7 | 71.3 (7.4) |  | 102 (5.0) | 6 | 71.6 (6.0) |  | 59 (4.2) | 7 | 75.7 (4.8) |
| Coronary heart disease & heart failure | 114 (4.3) | 8 | 71.6 (7.6) |  | 81 (4.0) | 8 | 73.4 (6.4) |  | 68 (4.9) | 6 | 77.1 (4.8) |

Abbreviation: SD, standard deviation.

^a^ % of the total number of incident multimorbidity cases (N=2,659 at age 50, N=2,029 at age 60, N=1,402 at age 70).
