# Supplementary material for: Association of sleep duration at age 50, 60, and 70 years with risk of multimorbidity in the UK: 25-year follow-up of the Whitehall II cohort study
Source: PLoS Med. 2022 Oct 18;19(10):e1004109. doi: 10.1371/journal.pmed.1004109 (PMC9578599; doi:10.1371/journal.pmed.1004109)
Supplement: S4 Table — (DOCX) [file pmed.1004109.s007.docx]

**S4 Table. Association** **of sleep duration at age 50, 60, and 70 with risk of multimorbidity^a^ among participants free from prevalent chronic disease**

|  | **N cases/  N total** | **Model 1: Unadjusted model (age as time-scale)** | | **Model 2:  Adjusted for socio-demographic variables^b^** | | **Model 3:  Model 2 + behavioral and  health-related factors^c^** | |
| --- | --- | --- | --- | --- | --- | --- | --- |
|  |  | HR (95%CI) | p-value | HR (95%CI) | p-value | HR (95%CI) | p-value |
| **Sleep duration  at age 50** | **N cases/N total = 2,329/7,217; Follow-up mean (SD)= 23.2 (7.2) years; mean age at event (SD)=71.9 (7.2) years** | | | | | | |
| ≤5 hours | 185/474 | 1.52 (1.30, 1.77) | <0.001 | 1.43 (1.22, 1.68) | <0.001 | 1.29 (1.10, 1.51) | 0.002 |
| 6 hours | 748/2,350 | 1.13 (1.03, 1.25) | 0.009 | 1.12 (1.01, 1.22) | 0.023 | 1.07 (0.98, 1.18) | 0.139 |
| 7 hours | 1,058/3,323 | 1.00 (ref) |  | 1.00 (ref) |  | 1.00 (ref) |  |
| 8 hours | 314/1,008 | 0.97 (0.86, 1.10) | 0.638 | 0.95 (0.84, 1.08) | 0.458 | 0.95 (0.84, 1.08) | 0.450 |
| ≥9 hours | 24/62 | 1.36 (0.91, 2.04) | 0.137 | 1.21 (0.80, 1.81) | 0.364 | 1.16 (0.77, 1.74) | 0.485 |
| **Sleep duration  at age 60** | **N cases/N total = 1,326/5,427; Follow-up mean (SD)= 14.3 (5.8) years; mean age at event (SD)=73.9 (5.9) years** | | | | | | |
| ≤5 hours | 124/377 | 1.51 (1.24, 1.84) | <0.001 | 1.42 (1.16, 1.73) | 0.001 | 1.31 (1.07, 1.60) | 0.009 |
| 6 hours | 424/1,660 | 1.13 (0.99, 1.28) | 0.065 | 1.11 (0.98, 1.26) | 0.107 | 1.07 (0.94, 1.22) | 0.287 |
| 7 hours | 535/2,316 | 1.00 (ref) |  | 1.00 (ref) |  | 1.00 (ref) |  |
| 8 hours | 212/980 | 0.95 (0.81, 1.11) | 0.498 | 0.94 (0.80, 1.10) | 0.434 | 0.92 (0.79, 1.08) | 0.320 |
| ≥9 hours | 31/94 | 1.60 (1.11, 2.30) | 0.011 | 1.58 (1.10, 2.27) | 0.014 | 1.57 (1.09, 2.27) | 0.015 |
| **Sleep duration  at age 70** | **N cases/N total = 661/3,648; Follow-up mean (SD)= 7.5 (4.6) years; mean age at event (SD)= 78.0 (4.5) years** | | | | | | |
| ≤5 hours | 60/257 | 1.44 (1.08, 1.90) | 0.012 | 1.41 (1.05, 1.88) | 0.021 | 1.38 (1.03, 1.85) | 0.032 |
| 6 hours | 196/1,013 | 1.16 (0.96, 1.40) | 0.115 | 1.15 (0.96, 1.39) | 0.135 | 1.14 (0.95, 1.38) | 0.159 |
| 7 hours | 254/1,524 | 1.00 (ref) |  | 1.00 (ref) |  | 1.00 (ref) |  |
| 8 hours | 129/770 | 1.06 (0.86, 1.31) | 0.569 | 1.05 (0.85, 1.30) | 0.650 | 1.02 (0.82, 1.26) | 0.868 |
| ≥9 hours | 22/84 | 1.55 (1.00, 2.39) | 0.050 | 1.54 (0.99, 2.38) | 0.054 | 1.52 (0.98, 2.35) | 0.063 |

Abbreviations: CI, confidence intervals; HR, hazard ratio; ref, reference; SD, standard deviation.

^a^ Multimorbidity defined as 2 or more of the following chronic diseases: diabetes, cancer, coronary heart disease, stroke, heart failure, chronic obstructive pulmonary disease, chronic kidney disease, liver disease, depression, dementia, other mental disorder, Parkinson’s disease, and arthritis/rheumatoid arthritis.

^b^ Adjusted for age (time-scale), sex, ethnicity, education, occupational position, and marital status.

^c^ Additionally adjusted for alcohol consumption, physical activity, smoking status, fruit and vegetable consumption, BMI, hypertension, and use of sleep medication.
