# Supplementary material for: Association of sleep duration at age 50, 60, and 70 years with risk of multimorbidity in the UK: 25-year follow-up of the Whitehall II cohort study
Source: PLoS Med. 2022 Oct 18;19(10):e1004109. doi: 10.1371/journal.pmed.1004109 (PMC9578599; doi:10.1371/journal.pmed.1004109)
Supplement: S5 Table — (DOCX) [file pmed.1004109.s008.docx]

**S5 Table. Association of sleep duration at age 50, 60, and 70 with risk of multimorbidity^a^ in analyses excluding participants on sleep medication**

|  | **N cases/  N total** | **Model 1: Unadjusted model (age as time-scale)** | | **Model 2:  Adjusted for socio-demographic variables^b^** | | **Model 3:  Model 2 + behavioral and  health-related factors^c^** | |
| --- | --- | --- | --- | --- | --- | --- | --- |
|  |  | HR (95%CI) | p-value | HR (95%CI) | p-value | HR (95%CI) | p-value |
| **Sleep duration  at age 50** | **N cases/N total = 2,601/7,757; Follow-up mean (SD) = 22.6 (7.5); mean age at event (SD) = 70.9 (7.7) years** | | | | | | |
| ≤5 hours | 216/524 | 1.58 (1.37, 1.83) | <0.001 | 1.48 (1.28, 1.71) | <0.001 | 1.30 (1.12, 1.51) | <0.001 |
| 6 hours | 823/2,515 | 1.11 (1.02, 1.22) | 0.018 | 1.09 (1.00, 1.20) | 0.047 | 1.06 (0.97, 1.16) | 0.220 |
| 7 hours | 1,168/3,556 | 1.00 (ref) |  | 1.00 (ref) |  | 1.00 (ref) |  |
| 8 hours | 362/1,087 | 1.01 (0.90, 1.14) | 0.855 | 0.99 (0.88, 1.12) | 0.879 | 0.99 (0.88, 1.11) | 0.846 |
| ≥9 hours | 32/75 | 1.58 (1.11, 2.25) | 0.011 | 1.41 (0.99, 2.00) | 0.059 | 1.37 (0.96, 1.96) | 0.079 |
| **Sleep duration  at age 60** | **N cases/N total = 2,007/6,793; Follow-up mean (SD) = 13.4 (6.0); mean age at event (SD) = 72.0 (6.3) years** | | | | | | |
| ≤5 hours | 197/504 | 1.59 (1.36, 1.86) | <0.001 | 1.47 (1.26, 1.73) | <0.001 | 1.35 (1.15, 1.58) | <0.001 |
| 6 hours | 635/2,073 | 1.15 (1.04, 1.28) | 0.008 | 1.13 (1.02, 1.25) | 0.023 | 1.13 (1.02, 1.25) | 0.023 |
| 7 hours | 789/2,869 | 1.00 (ref) |  | 1.00 (ref) |  | 1.00 (ref) |  |
| 8 hours | 338/1,226 | 1.03 (0.90, 1.17) | 0.688 | 1.02 (0.90, 1.16) | 0.747 | 1.06 (0.93, 1.20) | 0.386 |
| ≥9 hours | 48/121 | 1.62 (1.21, 2.17) | 0.001 | 1.58 (1.18, 2.12) | 0.002 | 1.53 (1.15, 2.06) | 0.004 |
| **Sleep duration  at age 70** | **N cases/N total = 1,389/5,499; Follow-up mean (SD) = 6.8 (4.5); mean age at event (SD) = 76.0 (4.8) years** | | | | | | |
| ≤5 hours | 150/438 | 1.68 (1.40, 2.02) | <0.001 | 1.64 (1.36, 1.98) | <0.001 | 1.42 (1.17, 1.71) | <0.001 |
| 6 hours | 417/1,556 | 1.18 (1.04, 1.34) | 0.012 | 1.17 (1.02, 1.33) | 0.022 | 1.10 (0.97, 1.26) | 0.142 |
| 7 hours | 512/2,237 | 1.00 (ref) |  | 1.00 (ref) |  | 1.00 (ref) |  |
| 8 hours | 268/1,147 | 1.04 (0.89, 1.20) | 0.646 | 1.03 (0.89, 1.19) | 0.701 | 0.98 (0.84, 1.13) | 0.767 |
| ≥9 hours | 42/121 | 1.51 (1.10, 2.07) | 0.010 | 1.48 (1.08, 2.02) | 0.016 | 1.50 (1.10, 2.07) | 0.012 |

Abbreviations: CI, confidence intervals; HR, hazard ratio; ref, reference; SD, standard deviation.

^a^ Multimorbidity defined as 2 or more of the following chronic diseases: diabetes, cancer, coronary heart disease, stroke, heart failure, chronic obstructive pulmonary disease, chronic kidney disease, liver disease, depression, dementia, other mental disorder, Parkinson’s disease, and arthritis/rheumatoid arthritis.

^b^ Adjusted for age (time-scale), sex, ethnicity, education, occupational position, and marital status.

^c^ Additionally adjusted for alcohol consumption, physical activity, smoking status, fruit and vegetable consumption, BMI, hypertension, and prevalence of one of the 13 chronic diseases.
