# Supplementary material for: Association of sleep duration at age 50, 60, and 70 years with risk of multimorbidity in the UK: 25-year follow-up of the Whitehall II cohort study
Source: PLoS Med. 2022 Oct 18;19(10):e1004109. doi: 10.1371/journal.pmed.1004109 (PMC9578599; doi:10.1371/journal.pmed.1004109)
Supplement: S6 Table — (DOCX) [file pmed.1004109.s009.docx]

**S6 Table. Association of sleep duration at age 50, 60, and 70 with risk of multimorbidity^a^ using inverse probability weighting analyses to take missing data into account**

|  | **N cases/  N total** | **Model 1: Unadjusted model (age as time-scale)** | | **Model 2:  Adjusted for socio-demographic variables^b^** | | **Model 3:  Model 2 + behavioral and  health-related factors^c^** | |
| --- | --- | --- | --- | --- | --- | --- | --- |
|  |  | HR (95%CI) | p-value | HR (95%CI) | p-value | HR (95%CI) | p-value |
| **Sleep duration  at age 50** | **N cases/N total = 2,659/7,864; Follow-up mean (SD) = 22.6 (7.5) years; mean age at event (SD) = 70.9 (7.7) years** | | | | | | |
| ≤5 hours | 225/544 | 1.64 (1.41, 1.92) | <0.001 | 1.53 (1.31, 1.79) | <0.001 | 1.34 (1.14, 1.57) | <0.001 |
| 6 hours | 852/2,562 | 1.13 (1.03, 1.24) | 0.008 | 1.11 (1.02, 1.22) | 0.020 | 1.07 (0.98, 1.18) | 0.131 |
| 7 hours | 1,184/3,589 | 1.00 (ref) |  | 1.00 (ref) |  | 1.00 (ref) |  |
| 8 hours | 365/1,092 | 1.01 (0.90, 1.14) | 0.865 | 0.99 (0.88, 1.12) | 0.876 | 1.00 (0.88, 1.13) | 0.967 |
| ≥9 hours | 33/77 | 1.67 (1.15, 2.43) | 0.007 | 1.49 (1.02, 2.19) | 0.042 | 1.46 (1.02, 2.09) | 0.039 |
| **Sleep duration  at age 60** | **N cases/N total = 2,029/6,848; Follow-up mean (SD)= 13.4 (6.0) years; mean age at event (SD) = 72.0 (6.3) years** | | | | | | |
| ≤5 hours | 202/519 | 1.57 (1.34, 1.83) | <0.001 | 1.46 (1.24, 1.70) | <0.001 | 1.33 (1.13, 1.58) | 0.001 |
| 6 hours | 645/2,095 | 1.13 (1.02, 1.26) | 0.023 | 1.11 (1.00, 1.24) | 0.057 | 1.11 (0.99, 1.24) | 0.071 |
| 7 hours | 793/2,882 | 1.00 (ref) |  | 1.00 (ref) |  | 1.00 (ref) |  |
| 8 hours | 340/1,230 | 1.06 (0.93, 1.20) | 0.408 | 1.05 (0.92, 1.20) | 0.454 | 1.07 (0.94, 1.23) | 0.302 |
| ≥9 hours | 49/122 | 1.59 (1.16, 2.17) | 0.004 | 1.56 (1.14, 2.13) | 0.006 | 1.54 (1.11, 2.14) | 0.010 |
| **Sleep duration  at age 70** | **N cases/N total = 1,402/5,546; Follow-up mean (SD)= 6.8 (4.5) years; mean age at event (SD) = 76.0 (4.8) years** | | | | | | |
| ≤5 hours | 152/451 | 1.61 (1.33, 1.94) | <0.001 | 1.53 (1.26, 1.87) | <0.001 | 1.36 (1.13, 1.65) | 0.001 |
| 6 hours | 425/1,574 | 1.17 (1.03, 1.34) | 0.018 | 1.15 (1.01, 1.32) | 0.038 | 1.09 (0.95, 1.26) | 0.223 |
| 7 hours | 514/2,249 | 1.00 (ref) |  | 1.00 (ref) |  | 1.00 (ref) |  |
| 8 hours | 269/1,151 | 1.06 (0.91, 1.23) | 0.486 | 1.05 (0.90, 1.22) | 0.549 | 1.00 (0.86, 1.17) | 0.971 |
| ≥9 hours | 42/121 | 1.52 (1.09, 2.11) | 0.013 | 1.50 (1.07, 2.10) | 0.018 | 1.57 (1.11, 2.22) | 0.011 |

Abbreviations: CI, confidence intervals; HR, hazard ratio; ref, reference; SD, standard deviation.

^a^ Multimorbidity defined as 2 or more of the following chronic diseases: diabetes, cancer, coronary heart disease, stroke, heart failure, chronic obstructive pulmonary disease, chronic kidney disease, liver disease, depression, dementia, other mental disorder, Parkinson’s disease, and arthritis/rheumatoid arthritis.

^b^ Adjusted for age (time-scale), sex, ethnicity, education, occupational position, and marital status.

^c^ Additionally adjusted for alcohol consumption, physical activity, smoking status, fruit and vegetable consumption, BMI, hypertension, use of sleep medication, and prevalence of one of the 13 chronic diseases.
