# Supplementary material for: Association of sleep duration at age 50, 60, and 70 years with risk of multimorbidity in the UK: 25-year follow-up of the Whitehall II cohort study
Source: PLoS Med. 2022 Oct 18;19(10):e1004109. doi: 10.1371/journal.pmed.1004109 (PMC9578599; doi:10.1371/journal.pmed.1004109)
Supplement: S7 Table — (DOCX) [file pmed.1004109.s010.docx]

**S7 Table. Association of sleep duration at age 50, 60, and 70 with risk of multimorbidity: impact of removing one chronic disease at a time from the list of chronic diseases included in the definition of multimorbidity**

| **Disease excluded from multimorbidity definition** |  | **Sleep duration  at age 50** | | | **Sleep duration  at age 60** | | **Sleep duration  at age 70** | |
| --- | --- | --- | --- | --- | --- | --- | --- | --- |
|  | **Sleep duration** | | HR^a^ (95%CI) | p-value | HR^a^ (95%CI) | p-value | HR^a^ (95%CI) | p-value |
| **Diabetes** |  | |  |  |  |  |  |  |
|  | ≤5 hours | | 1.31 (1.12, 1.53) | 0.001 | 1.33 (1.12, 1.58) | 0.001 | 1.43 (1.17, 1.74) | <0.001 |
|  | 6 hours | | 1.13 (1.02, 1.24) | 0.015 | 1.11 (0.99, 1.25) | 0.065 | 1.18 (1.02, 1.35) | 0.022 |
|  | 7 hours | | 1.00 (ref) |  | 1.00 (ref) |  | 1.00 (ref) |  |
|  | 8 hours | | 1.03 (0.91, 1.17) | 0.641 | 1.07 (0.93, 1.22) | 0.364 | 0.99 (0.84, 1.16) | 0.901 |
|  | ≥9 hours | | 1.44 (0.99, 2.08) | 0.057 | 1.22 (0.87, 1.69) | 0.249 | 1.50 (1.07, 2.11) | 0.019 |
| **Cancer** | | |  |  |  |  |  |  |
|  | ≤5 hours | | 1.35 (1.15, 1.58) | <0.001 | 1.35 (1.13, 1.61) | 0.001 | 1.37 (1.11, 1.69) | 0.004 |
|  | 6 hours | | 1.09 (0.99, 1.21) | 0.071 | 1.19 (1.06, 1.34) | 0.004 | 1.21 (1.05, 1.40) | 0.008 |
|  | 7 hours | | 1.00 (ref) |  | 1.00 (ref) |  | 1.00 (ref) |  |
|  | 8 hours | | 1.06 (0.93, 1.21) | 0.366 | 1.12 (0.97, 1.29) | 0.119 | 0.97 (0.82, 1.16) | 0.770 |
|  | ≥9 hours | | 1.42 (0.97, 2.08) | 0.068 | 1.37 (0.99, 1.90) | 0.057 | 1.83 (1.31, 2.56) | <0.001 |
| **Coronary heart disease** | | |  |  |  |  |  |  |
|  | ≤5 hours | | 1.31 (1.12, 1.54) | 0.001 | 1.25 (1.05, 1.50) | 0.014 | 1.36 (1.11, 1.67) | 0.003 |
|  | 6 hours | | 1.09 (0.99, 1.20) | 0.094 | 1.10 (0.98, 1.24) | 0.103 | 1.02 (0.88, 1.17) | 0.834 |
|  | 7 hours | | 1.00 (ref) |  | 1.00 (ref) |  | 1.00 (ref) |  |
|  | 8 hours | | 1.06 (0.93, 1.21) | 0.362 | 1.11 (0.96, 1.28) | 0.170 | 0.97 (0.82, 1.14) | 0.679 |
|  | ≥9 hours | | 1.46 (1.00, 2.13) | 0.051 | 1.71 (1.25, 2.34) | 0.001 | 1.22 (0.85, 1.75) | 0.292 |
| **Stroke** |  | |  |  |  |  |  |  |
|  | ≤5 hours | | 1.29 (1.12, 1.50) | 0.001 | 1.36 (1.15, 1.59) | <0.001 | 1.42 (1.18, 1.72) | <0.001 |
|  | 6 hours | | 1.09 (0.99, 1.19) | 0.070 | 1.18 (1.06, 1.32) | 0.002 | 1.15 (1.01, 1.32) | 0.036 |
|  | 7 hours | | 1.00 (ref) |  | 1.00 (ref) |  | 1.00 (ref) |  |
|  | 8 hours | | 0.97 (0.86, 1.10) | 0.683 | 1.06 (0.93, 1.21) | 0.368 | 1.01 (0.87, 1.17) | 0.916 |
|  | ≥9 hours | | 1.31 (0.91, 1.87) | 0.146 | 1.61 (1.20, 2.16) | 0.001 | 1.46 (1.05, 2.04) | 0.026 |
| **Heart failure** |  | |  |  |  |  |  |  |
|  | ≤5 hours | | 1.31 (1.13, 1.52) | <0.001 | 1.30 (1.11, 1.53) | 0.001 | 1.30 (1.07, 1.58) | 0.008 |
|  | 6 hours | | 1.07 (0.98, 1.17) | 0.157 | 1.13 (1.02, 1.26) | 0.020 | 1.10 (0.96, 1.26) | 0.154 |
|  | 7 hours | | 1.00 (ref) |  | 1.00 (ref) |  | 1.00 (ref) |  |
|  | 8 hours | | 0.99 (0.88, 1.11) | 0.839 | 1.06 (0.93, 1.20) | 0.402 | 0.97 (0.83, 1.13) | 0.674 |
|  | ≥9 hours | | 1.40 (0.98, 2.00) | 0.063 | 1.51 (1.12, 2.03) | 0.006 | 1.52 (1.11, 2.10) | 0.010 |

**S7 Table (Continued).**

| **Disease excluded from multimorbidity definition** |  | **Sleep duration  at age 50** | | **Sleep duration  at age 60** | | **Sleep duration  at age 70** | |
| --- | --- | --- | --- | --- | --- | --- | --- |
|  | **Sleep duration** | HR^a^ (95%CI) | p-value | HR^a^ (95%CI) | p-value | HR^a^ (95%CI) | p-value |
| **Chronic obstructive pulmonary disease** |  |  |  |  |  |  |  |
|  | ≤5 hours | 1.30 (1.13, 1.51) | <0.001 | 1.39 (1.18, 1.63) | <0.001 | 1.48 (1.23, 1.79) | <0.001 |
|  | 6 hours | 1.07 (0.98, 1.17) | 0.149 | 1.17 (1.05, 1.30) | 0.004 | 1.16 (1.02, 1.33) | 0.026 |
|  | 7 hours | 1.00 (ref) |  | 1.00 (ref) |  | 1.00 (ref) |  |
|  | 8 hours | 0.99 (0.88, 1.12) | 0.878 | 1.07 (0.94, 1.22) | 0.304 | 0.99 (0.85, 1.15) | 0.878 |
|  | ≥9 hours | 1.42 (1.00, 2.01) | 0.049 | 1.61 (1.21, 2.16) | 0.001 | 1.47 (1.06, 2.04) | 0.021 |
| **Chronic kidney disease** | |  |  |  |  |  |  |
|  | ≤5 hours | 1.34 (1.16, 1.56) | <0.001 | 1.34 (1.14, 1.57) | <0.001 | 1.45 (1.20, 1.75) | <0.001 |
|  | 6 hours | 1.08 (0.99, 1.19) | 0.076 | 1.15 (1.04, 1.28) | 0.008 | 1.17 (1.02, 1.33) | 0.024 |
|  | 7 hours | 1.00 (ref) |  | 1.00 (ref) |  | 1.00 (ref) |  |
|  | 8 hours | 1.00 (0.89, 1.13) | 0.972 | 1.07 (0.94, 1.22) | 0.295 | 1.01 (0.86, 1.17) | 0.932 |
|  | ≥9 hours | 1.41 (0.99, 2.01) | 0.055 | 1.45 (1.07, 1.96) | 0.016 | 1.37 (0.99, 1.91) | 0.061 |
| **Liver disease** |  |  |  |  |  |  |  |
|  | ≤5 hours | 1.30 (1.12, 1.50) | <0.001 | 1.33 (1.13, 1.56) | <0.001 | 1.44 (1.19, 1.73) | <0.001 |
|  | 6 hours | 1.09 (1.00, 1.19) | 0.056 | 1.15 (1.03, 1.28) | 0.011 | 1.12 (0.98, 1.28) | 0.097 |
|  | 7 hours | 1.00 (ref) |  | 1.00 (ref) |  | 1.00 (ref) |  |
|  | 8 hours | 1.00 (0.89, 1.13) | 0.986 | 1.04 (0.92, 1.19) | 0.528 | 1.01 (0.87, 1.17) | 0.947 |
|  | ≥9 hours | 1.35 (0.95, 1.93) | 0.093 | 1.55 (1.16, 2.08) | 0.003 | 1.52 (1.10, 2.10) | 0.011 |
| **Depression** |  |  |  |  |  |  |  |
|  | ≤5 hours | 1.27 (1.09, 1.48) | 0.002 | 1.35 (1.14, 1.59) | <0.001 | 1.41 (1.16, 1.71) | 0.001 |
|  | 6 hours | 1.07 (0.97, 1.18) | 0.153 | 1.13 (1.01, 1.26) | 0.034 | 1.12 (0.97, 1.28) | 0.111 |
|  | 7 hours | 1.00 (ref) |  | 1.00 (ref) |  | 1.00 (ref) |  |
|  | 8 hours | 1.02 (0.90, 1.15) | 0.737 | 1.04 (0.91, 1.19) | 0.557 | 0.99 (0.85, 1.16) | 0.924 |
|  | ≥9 hours | 1.32 (0.92, 1.89) | 0.134 | 1.51 (1.10, 2.05) | 0.010 | 1.42 (1.02, 1.98) | 0.040 |
| **Dementia** |  |  |  |  |  |  |  |
|  | ≤5 hours | 1.26 (1.09, 1.46) | 0.002 | 1.34 (1.14, 1.57) | <0.001 | 1.40 (1.16, 1.70) | 0.001 |
|  | 6 hours | 1.05 (0.96, 1.15) | 0.322 | 1.11 (0.99, 1.23) | 0.062 | 1.13 (0.99, 1.29) | 0.081 |
|  | 7 hours | 1.00 (ref) |  | 1.00 (ref) |  | 1.00 (ref) |  |
|  | 8 hours | 0.98 (0.87, 1.11) | 0.738 | 1.07 (0.94, 1.22) | 0.330 | 0.97 (0.83, 1.13) | 0.722 |
|  | ≥9 hours | 1.44 (1.02, 2.05) | 0.039 | 1.63 (1.22, 2.17) | 0.001 | 1.51 (1.09, 2.09) | 0.014 |

**S7 Table (Continued).**

| **Disease excluded from multimorbidity definition** |  | **Sleep duration  at age 50** | | | **Sleep duration  at age 60** | | **Sleep duration  at age 70** | |
| --- | --- | --- | --- | --- | --- | --- | --- | --- |
|  | **Sleep duration** | | HR^a^ (95%CI) | p-value | HR^a^ (95%CI) | p-value | HR^a^ (95%CI) | p-value |
| **Other mental disorder** |  | |  |  |  |  |  |  |
|  | ≤5 hours | | 1.33 (1.15, 1.54) | <0.001 | 1.30 (1.11, 1.53) | 0.001 | 1.39 (1.15, 1.68) | 0.001 |
|  | 6 hours | | 1.07 (0.98, 1.18) | 0.118 | 1.13 (1.01, 1.26) | 0.026 | 1.11 (0.97, 1.27) | 0.118 |
|  | 7 hours | | 1.00 (ref) |  | 1.00 (ref) |  | 1.00 (ref) |  |
|  | 8 hours | | 1.00 (0.89, 1.13) | 0.972 | 1.05 (0.92, 1.19) | 0.490 | 0.99 (0.85, 1.16) | 0.931 |
|  | ≥9 hours | | 1.42 (1.00, 2.02) | 0.052 | 1.48 (1.10, 2.00) | 0.010 | 1.58 (1.15, 2.17) | 0.005 |
| **Parkinson’s disease** |  | |  |  |  |  |  |  |
|  | ≤5 hours | | 1.31 (1.13, 1.51) | <0.001 | 1.27 (1.08, 1.49) | 0.003 | 1.36 (1.12, 1.64) | 0.002 |
|  | 6 hours | | 1.08 (0.98, 1.18) | 0.104 | 1.16 (1.04, 1.28) | 0.007 | 1.11 (0.98, 1.27) | 0.104 |
|  | 7 hours | | 1.00 (ref) |  | 1.00 (ref) |  | 1.00 (ref) |  |
|  | 8 hours | | 1.00 (0.89, 1.12) | 0.964 | 1.06 (0.93, 1.20) | 0.388 | 0.97 (0.84, 1.13) | 0.690 |
|  | ≥9 hours | | 1.36 (0.96, 1.94) | 0.087 | 1.58 (1.18, 2.11) | 0.002 | 1.50 (1.08, 2.06) | 0.014 |
| **Arthritis/rheumatoid arthritis** |  | |  |  |  |  |  |  |
|  | ≤5 hours | | 1.22 (1.05, 1.43) | 0.011 | 1.34 (1.13, 1.59) | 0.001 | 1.35 (1.10, 1.66) | 0.004 |
|  | 6 hours | | 1.04 (0.94, 1.14) | 0.448 | 1.11 (0.99, 1.24) | 0.079 | 1.04 (0.90, 1.20) | 0.604 |
|  | 7 hours | | 1.00 (ref) |  | 1.00 (ref) |  | 1.00 (ref) |  |
|  | 8 hours | | 1.01 (0.89, 1.14) | 0.894 | 1.09 (0.95, 1.25) | 0.219 | 0.97 (0.82, 1.14) | 0.696 |
|  | ≥9 hours | | 1.30 (0.89, 1.89) | 0.178 | 1.57 (1.14, 2.14) | 0.005 | 1.59 (1.13, 2.24) | 0.008 |

Abbreviations: CI, confidence intervals; HR, hazard ratio; ref, reference; SD, standard deviation.

^a^ Adjusted for age (time-scale), sex, ethnicity, education, occupational position, marital status, alcohol consumption, physical activity, smoking status, fruit and vegetable consumption, BMI, hypertension, use of sleep medication, and prevalence of one of the 13 chronic diseases.
