# Supplementary material for: Association of sleep duration at age 50, 60, and 70 years with risk of multimorbidity in the UK: 25-year follow-up of the Whitehall II cohort study
Source: PLoS Med. 2022 Oct 18;19(10):e1004109. doi: 10.1371/journal.pmed.1004109 (PMC9578599; doi:10.1371/journal.pmed.1004109)
Supplement: S8 Table — (DOCX) [file pmed.1004109.s011.docx]

**S8 Table. Description of sleep duration at age 50, 60, and 70 by groups of trajectories of sleep duration between age 50 and 70.**

|  |  | **Sleep duration**  Mean (standard deviation), hours | | |
| --- | --- | --- | --- | --- |
| **Trajectories of sleep duration between age 50 and 70** | N | **Age 50** | **Age 60** | **Age 70** |
| Persistent short | 1,034 | 5.8 (0.4) | 5.7 (0.4) | 5.7 (0.5) |
| Persistent normal | 2,074 | 7.2 (0.4) | 7.0 (0.6) | 6.8 (0.5) |
| Persistent long | 368 | 8.1 (0.2) | 8.1 (0.3) | 8.0 (0.6) |
| Change from short to normal | 903 | 5.9 (0.3) | 6.6 (0.6) | 6.9 (0.6) |
| Change from normal to long | 769 | 6.9 (0.5) | 7.5 (0.6) | 8.1 (0.3) |
| Change from normal to short | 362 | 7.1 (0.3) | 5.9 (0.4) | 5.8 (0.4) |
