# Supplementary material for: Association of sleep duration at age 50, 60, and 70 years with risk of multimorbidity in the UK: 25-year follow-up of the Whitehall II cohort study
Source: PLoS Med. 2022 Oct 18;19(10):e1004109. doi: 10.1371/journal.pmed.1004109 (PMC9578599; doi:10.1371/journal.pmed.1004109)
Supplement: S9 Table — (DOCX) [file pmed.1004109.s012.docx]

**S9 Table.** **Association of trajectories of sleep duration between age 50 and 70 with risk of multimorbidity^a^**

|  | **N cases/  N total** | **Model 1: Unadjusted model (age as time-scale)** | | **Model 2:  Adjusted for socio-demographic variables^b^** | | **Model 3:  Model 2 + behavioral and health-related factors^c^** | |
| --- | --- | --- | --- | --- | --- | --- | --- |
|  |  | HR (95%CI) | p-value | HR (95%CI) | p-value | HR (95%CI) | p-value |
| **Trajectories of sleep duration between age 50 and 70** | **N cases/N total = 1,419/5,510; Follow-up mean (SD) = 6.8 (4.5) years; mean age at event (SD) = 76.0 (4.8) years** | | | | | | |
| Persistent short | 295/1,034 | 1.26 (1.09, 1.45) | 0.002 | 1.23 (1.06, 1.42) | 0.006 | 1.17 (1.01, 1.35) | 0.040 |
| Persistent normal | 515/2,074 | 1.00 (ref) |  | 1.00 (ref) |  | 1.00 (ref) |  |
| Persistent long | 96/368 | 1.03 (0.83, 1.28) | 0.796 | 1.03 (0.83, 1.28) | 0.802 | 1.06 (0.85, 1.32) | 0.608 |
| Change from short to normal | 217/903 | 1.12 (0.95, 1.31) | 0.181 | 1.11 (0.95, 1.31) | 0.185 | 1.09 (0.93, 1.28) | 0.274 |
| Change from normal to long | 179/769 | 1.03 (0.87, 1.22) | 0.755 | 1.02 (0.86, 1.20) | 0.859 | 0.99 (0.84, 1.18) | 0.927 |
| Change from normal to short | 117/362 | 1.17 (0.96, 1.43) | 0.121 | 1.16 (0.95, 1.42) | 0.154 | 1.13 (0.92, 1.39) | 0.229 |

Abbreviations: CI, confidence intervals; HR, hazard ratio; ref, reference; SD, standard deviation.

^a^ Multimorbidity defined as 2 or more of the following chronic diseases: diabetes, cancer, coronary heart disease, stroke, heart failure, chronic obstructive pulmonary disease, chronic kidney disease, liver disease, depression, dementia, other mental disorder, Parkinson’s disease, and arthritis/rheumatoid arthritis.

^b^ Adjusted for age (time-scale), sex, ethnicity, education, occupational position, and marital status.

^c^ Additionally adjusted for alcohol consumption, physical activity, smoking status, fruit and vegetable consumption, BMI, hypertension, use of sleep medication, and prevalence of one of the 13 chronic diseases.
