# Supplementary material for: Association of sleep duration at age 50, 60, and 70 years with risk of multimorbidity in the UK: 25-year follow-up of the Whitehall II cohort study
Source: PLoS Med. 2022 Oct 18;19(10):e1004109. doi: 10.1371/journal.pmed.1004109 (PMC9578599; doi:10.1371/journal.pmed.1004109)
Supplement: S10 Table — (DOCX) [file pmed.1004109.s013.docx]

**S10 Table. Sleep disturbances assessed using the Jenkins sleep problems scale as a function of sleep duration**

| **Sleep duration** | **N** | **Mean (SD) Jenkins sleep problems score** | **p-value** |
| --- | --- | --- | --- |
| **At age 60** |  |  |  |
| ≤5 hours | 481 | 10.1 (5.7) | <0.001 |
| 6 hours | 1,949 | 6.3 (4.6) | <0.001 |
| 7 hours | 2,672 | 4.4 (3.5) | ref |
| 8 hours | 1,166 | 3.6 (3.4) | <0.001 |
| ≥9 hours | 113 | 4.4 (4.1) | 0.925 |
| **At age 70** |  |  |  |
| ≤5 hours | 438 | 10.6 (5.4) | <0.001 |
| 6 hours | 1,551 | 6.5 (4.5) | <0.001 |
| 7 hours | 2,219 | 4.5 (3.6) | ref |
| 8 hours | 1,137 | 3.7 (3.3) | <0.001 |
| ≥9 hours | 120 | 4.8 (4.3) | 0.378 |

Abbreviations: SD, standard deviation; ref, reference.
