# Supplementary material for: Association of sleep duration at age 50, 60, and 70 years with risk of multimorbidity in the UK: 25-year follow-up of the Whitehall II cohort study
Source: PLoS Med. 2022 Oct 18;19(10):e1004109. doi: 10.1371/journal.pmed.1004109 (PMC9578599; doi:10.1371/journal.pmed.1004109)
Supplement: S11 Table — (DOCX) [file pmed.1004109.s014.docx]

**S11 Table. Association of the Jenkins sleep problems scale with risk of multimorbidity^a^**

|  | **N cases/N total** | **Model 1: Unadjusted model (age as time-scale)** | | **Model 2:  Adjusted for socio-demographic variables^b^** | | **Model 3:  Model 2 + behavioral and health-related factors^c^** | |
| --- | --- | --- | --- | --- | --- | --- | --- |
|  |  | HR (95%CI) | p-value | HR (95%CI) | p-value | HR (95%CI) | p-value |
| **Jenkins sleep problems score at age 60** | **N cases/N total = 1,783/6,381; Follow-up mean (SD) = 12.8 (5.4) years; mean age at event (SD) = 71.3 (6.0) years** | | | | | | |
| Per 1-point increase | 1,783/6,381 | 1.04 (1.03, 1.05) | <0.001 | 1.04 (1.03, 1.05) | <0.001 | 1.03 (1.02, 1.04) | <0.001 |
| Low disturbance (0-11) | 1,542/5,710 | 1.00 (ref) |  | 1.00 (ref) |  | 1.00 (ref) |  |
| High disturbance (12-20) | 241/671 | 1.50 (1.31, 1.72) | <0.001 | 1.46 (1.27, 1.67) | <0.001 | 1.26 (1.10, 1.45) | 0.001 |
| **Jenkins sleep problems score at age 70** | **N cases/N total = 1,365/5,465; Follow-up mean (SD) = 6.8 (4.5) years; mean age at event (SD) = 76.0 (4.8) years** | | | | | | |
| Per 1-point increase | 1,365/5,465 | 1.04 (1.03, 1.05) | <0.001 | 1.04 (1.03, 1.05) | <0.001 | 1.03 (1.02, 1.04) | <0.001 |
| Low disturbance (0-11) | 1,187/4,884 | 1.00 (ref) |  | 1.00 (ref) |  | 1.00 (ref) |  |
| High disturbance (12-20) | 178/581 | 1.38 (1.18, 1.61) | <0.001 | 1.39 (1.19, 1.63) | <0.001 | 1.24 (1.05, 1.46) | 0.009 |

Abbreviations: CI, confidence intervals; HR, hazard ratio; ref, reference; SD, standard deviation.

^a^ Multimorbidity defined as 2 or more of the following chronic diseases: diabetes, cancer, coronary heart disease, stroke, heart failure, chronic obstructive pulmonary disease, chronic kidney disease, liver disease, depression, dementia, other mental disorder, Parkinson’s disease, and arthritis/rheumatoid arthritis.

^b^ Adjusted for age (time-scale), sex, ethnicity, education, occupational position, and marital status.

^c^ Additionally adjusted for alcohol consumption, physical activity, smoking status, fruit and vegetable consumption, BMI, hypertension, use of sleep medication, and prevalence of one of the 13 chronic diseases.
