# Supplementary material for: Association of sleep duration at age 50, 60, and 70 years with risk of multimorbidity in the UK: 25-year follow-up of the Whitehall II cohort study
Source: PLoS Med. 2022 Oct 18;19(10):e1004109. doi: 10.1371/journal.pmed.1004109 (PMC9578599; doi:10.1371/journal.pmed.1004109)
Supplement: S12 Table — (DOCX) [file pmed.1004109.s015.docx]

**S12 Table. Association of sleep duration at age 50 with transitions from a healthy state to first chronic disease, multimorbidity, and mortality (N=7,217) using inverse probability weighting analyses to take missing data into account**

| **Sleep duration at 50y** | **N cases/  N total** | **Model 1: Unadjusted model (age as time-scale)** | | **Model 2:  Adjusted for socio-demographic variables^a^** | | **Model 3:  Model 2 + behavioral and  health-related factors^b^** | |
| --- | --- | --- | --- | --- | --- | --- | --- |
|  |  | HR (95%CI) | p-value | HR (95%CI) | p-value | HR (95%CI) | p-value |
| **Healthy to First chronic disease^c^, transition A (N cases/N total = 4,446/7,217; mean age at event (SD) = 66.2 (8.5) years)** | | | | | | | |
| ≤5 hours | 319/474 | 1.36 (1.20, 1.54) | <0.001 | 1.33 (1.17, 1.51) | <0.001 | 1.24 (1.09, 1.40) | 0.001 |
| 6 hours | 1,428/2,350 | 1.06 (0.99, 1.14) | 0.089 | 1.05 (0.98, 1.13) | 0.141 | 1.02 (0.95, 1.10) | 0.539 |
| 7 hours | 2,038/3,323 | 1.00 (ref) |  | 1.00 (ref) |  | 1.00 (ref) |  |
| 8 hours | 621/1,008 | 0.99 (0.91, 1.09) | 0.897 | 0.98 (0.90, 1.08) | 0.719 | 0.98 (0.89, 1.08) | 0.673 |
| ≥9 hours | 40/62 | 1.13 (0.82, 1.57) | 0.457 | 1.08 (0.78, 1.49) | 0.659 | 1.08 (0.78, 1.49) | 0.644 |
| **Healthy to Death, transition B (N cases/N total = 213/7,217; mean age at event (SD) = 65.2 (9.0) years)** | | | | | | | |
| ≤5 hours | 14/474 | 1.25 (0.71, 2.23) | 0.442 | 1.13 (0.64, 2.01) | 0.674 | 0.97 (0.55, 1.72) | 0.918 |
| 6 hours | 71/2,350 | 1.17 (0.85, 1.62) | 0.336 | 1.12 (0.82, 1.55) | 0.477 | 1.05 (0.76, 1.46) | 0.749 |
| 7 hours | 98/3,323 | 1.00 (ref) |  | 1.00 (ref) |  | 1.00 (ref) |  |
| 8 hours | 29/1,008 | 0.98 (0.64, 1.50) | 0.931 | 0.96 (0.63, 1.46) | 0.843 | 0.96 (0.63, 1.46) | 0.834 |
| ≥9 hours | 1/62 | na |  | na |  | na |  |
| **First chronic disease^c^ to multimorbidity^c^, transition C (N cases/N total = 2,297/4,446; mean age at event (SD) = 71.9 (7.2) years)** | | | | | | | |
| ≤5 hours | 183/319 | 1.35 (1.13, 1.61) | 0.001 | 1.28 (1.07, 1.54) | 0.006 | 1.19 (0.99, 1.42) | 0.063 |
| 6 hours | 739/1,428 | 1.13 (1.02, 1.25) | 0.022 | 1.13 (1.02, 1.25) | 0.024 | 1.11 (1.00, 1.23) | 0.048 |
| 7 hours | 1,042/2,038 | 1.00 (ref) |  | 1.00 (ref) |  | 1.00 (ref) |  |
| 8 hours | 310/621 | 0.98 (0.85, 1.12) | 0.737 | 0.98 (0.85, 1.13) | 0.782 | 0.98 (0.85, 1.13) | 0.783 |
| ≥9 hours | 23/40 | 1.45 (0.86, 2.43) | 0.162 | 1.33 (0.78, 2.27) | 0.295 | 1.24 (0.73, 2.11) | 0.431 |
| **First chronic disease^c^ to Death, transition D (N cases/N total = 474/4,446; mean age at event (SD) = 68.9 (7.9) years)** | | | | | | | |
| ≤5 hours | 32/319 | 1.09 (0.72, 1.65) | 0.680 | 1.08 (0.70, 1.65) | 0.729 | 1.11 (0.72, 1.71) | 0.633 |
| 6 hours | 163/1,428 | 1.21 (0.97, 1.52) | 0.096 | 1.22 (0.97, 1.53) | 0.088 | 1.24 (0.99, 1.57) | 0.064 |
| 7 hours | 205/2,038 | 1.00 (ref) |  | 1.00 (ref) |  | 1.00 (ref) |  |
| 8 hours | 69/621 | 1.12 (0.83, 1.50) | 0.450 | 1.13 (0.84, 1.52) | 0.428 | 1.12 (0.83, 1.51) | 0.448 |
| ≥9 hours | 5/40 | na |  | na |  | na |  |
| **Multimorbidity^c^ to Death, transition E (N cases/N total = 787/2,297; mean age at event (SD) = 76.0 (6.8) years)** | | | | | | | |
| ≤5 hours | 65/183 | 1.08 (0.79, 1.48) | 0.641 | 1.11 (0.81, 1.51) | 0.530 | 1.10 (0.81, 1.50) | 0.537 |
| 6 hours | 254/739 | 1.11 (0.93, 1.33) | 0.246 | 1.12 (0.94, 1.34) | 0.210 | 1.13 (0.94, 1.36) | 0.189 |
| 7 hours | 348/1,042 | 1.00 (ref) |  | 1.00 (ref) |  | 1.00 (ref) |  |
| 8 hours | 112/310 | 1.09 (0.86, 1.37) | 0.473 | 1.11 (0.88, 1.40) | 0.364 | 1.11 (0.88, 1.39) | 0.377 |
| ≥9 hours | 8/23 | 1.05 (0.59, 1.85) | 0.879 | 1.19 (0.67, 2.12) | 0.562 | 1.22 (0.64, 2.34) | 0.543 |

Abbreviations: CI, confidence intervals; HR, hazard ratio; na, not applicable (N cases≤5); ref, reference; SD, standard deviation. For transitions see Fig 1

^a^ Adjusted for age (time-scale), sex, ethnicity, education, occupational position, and marital status.

^b^ Additionally adjusted for alcohol consumption, physical activity, smoking status, fruit and vegetable consumption, BMI, hypertension, and use of sleep medication.

^c^ Chronic disease among diabetes, cancer, coronary heart disease, stroke, heart failure, chronic obstructive pulmonary disease, chronic kidney disease, liver disease, depression, dementia, other mental disorder, Parkinson’s disease, and arthritis/rheumatoid arthritis; Multimorbidity defined as 2 or more of these diseases.
