# Supplementary material for: Association of sleep duration at age 50, 60, and 70 years with risk of multimorbidity in the UK: 25-year follow-up of the Whitehall II cohort study
Source: PLoS Med. 2022 Oct 18;19(10):e1004109. doi: 10.1371/journal.pmed.1004109 (PMC9578599; doi:10.1371/journal.pmed.1004109)
Supplement: S13 Table — (DOCX) [file pmed.1004109.s016.docx]

**S13 Table. Association of sleep duration at age 50 with risk of mortality^a^**

|  | **N cases/  N total** | **Model 1: Unadjusted model (age as time-scale)** | | **Model 2:  Adjusted for socio-demographic variables^b^** | | **Model 3:  Model 2 + behavioral and  health-related factors^c^** | |
| --- | --- | --- | --- | --- | --- | --- | --- |
|  |  | HR (95%CI) | p-value | HR (95%CI) | p-value | HR (95%CI) | p-value |
| **Sleep duration  at age 50** | **N cases/N total = 1,474/7,217; Follow-up mean (SD) = 25.2 (6.9) years; mean age at event (SD) = 72.2 (8.6) years** | | | | | | |
| ≤5 hours | 111/474 | 1.38 (1.13, 1.69) | 0.002 | 1.34 (1.10, 1.64) | 0.005 | 1.25 (1.02, 1.53) | 0.034 |
| 6 hours | 488/2,350 | 1.22 (1.08, 1.37) | 0.001 | 1.20 (1.07, 1.35) | 0.002 | 1.17 (1.04, 1.32) | 0.008 |
| 7 hours | 651/3,323 | 1.00 (ref) |  | 1.00 (ref) |  | 1.00 (ref) |  |
| 8 hours | 210/1,008 | 1.05 (0.90, 1.23) | 0.501 | 1.06 (0.91, 1.24) | 0.461 | 1.07 (0.92, 1.25) | 0.382 |
| ≥9 hours | 14/62 | 1.27 (0.75, 2.15) | 0.380 | 1.29 (0.76, 2.19) | 0.354 | 1.33 (0.78, 2.28) | 0.289 |

Abbreviations: CI, confidence intervals; HR, hazard ratio; ref, reference; SD, standard deviation.

^a^Among participants free of the 13 chronic diseases at age 50.

^b^Adjusted for age (time-scale), sex, ethnicity, education, occupational position, and marital status.

^c^Additionally adjusted for alcohol consumption, physical activity, smoking status, fruit and vegetable consumption, BMI, hypertension, and use of sleep medication.
