# Supplementary material for: Association of sleep duration at age 50, 60, and 70 years with risk of multimorbidity in the UK: 25-year follow-up of the Whitehall II cohort study
Source: PLoS Med. 2022 Oct 18;19(10):e1004109. doi: 10.1371/journal.pmed.1004109 (PMC9578599; doi:10.1371/journal.pmed.1004109)
Supplement: S14 Table — (DOCX) [file pmed.1004109.s017.docx]

**S14 Table. Association of sleep duration after first chronic disease with transitions to multimorbidity, and mortality in analyses using inverse probability weighting analyses to take missing data into account**

| **Sleep duration after first chronic disease** | **N cases/  N total** | **Model 1: Unadjusted model (age as time-scale)** | | **Model 2:  Adjusted for socio-demographic variables^a^** | | **Model 3:  Model 2 + behavioral and  health-related factors^b^** | |
| --- | --- | --- | --- | --- | --- | --- | --- |
|  |  | HR (95%CI) | p-value | HR (95%CI) | p-value | HR (95%CI) | p-value |
| **First chronic disease^c^ to multimorbidity^c^ (mean age at event (SD) = 70.1 (7.9) years)** | | | | | | | |
| ≤5 hours | 226/385 | 1.34 (1.15, 1.56) | <0.001 | 1.26 (1.08, 1.48) | 0.004 | 1.21 (1.04, 1.42) | 0.017 |
| 6 hours | 588/1,125 | 1.11 (0.99, 1.24) | 0.068 | 1.09 (0.98, 1.22) | 0.111 | 1.07 (0.96, 1.20) | 0.209 |
| 7 hours | 713/1,440 | 1.00 (ref) |  | 1.00 (ref) |  | 1.00 (ref) |  |
| 8 hours | 353/685 | 1.09 (0.96, 1.24) | 0.168 | 1.09 (0.96, 1.23) | 0.208 | 1.08 (0.95, 1.23) | 0.231 |
| ≥9 hours | 43/67 | 1.34 (0.97, 1.86) | 0.071 | 1.32 (0.95, 1.83) | 0.100 | 1.24 (0.89, 1.74) | 0.198 |
| **First chronic disease^c^ to Death (mean age at event (SD) = 69.7 (8.5) years)** | | | | | | | |
| ≤5 hours | 20/385 | 1.21 (0.73, 2.02) | 0.454 | 1.17 (0.69, 1.98) | 0.552 | 1.18 (0.70, 2.01) | 0.539 |
| 6 hours | 58/1,125 | 1.11 (0.79, 1.58) | 0.547 | 1.09 (0.77, 1.54) | 0.644 | 1.07 (0.75, 1.51) | 0.721 |
| 7 hours | 68/1,440 | 1.00 (ref) |  | 1.00 (ref) |  | 1.00 (ref) |  |
| 8 hours | 40/685 | 1.33 (0.90, 1.97) | 0.158 | 1.34 (0.90, 2.00) | 0.147 | 1.37 (0.92, 2.04) | 0.122 |
| ≥9 hours | 4/67 | na |  | na | 0.603 | na |  |
| **Multimorbidity^c^ to Death (mean age at event (SD) = 74.9 (7.5) years)** | | | | | | | |
| ≤5 hours | 63/226 | 1.04 (0.76, 1.42) | 0.813 | 1.10 (0.81, 1.50) | 0.532 | 1.14 (0.84, 1.55) | 0.400 |
| 6 hours | 158/588 | 1.00 (0.81, 1.25) | 0.977 | 1.05 (0.84, 1.30) | 0.679 | 1.02 (0.82, 1.28) | 0.827 |
| 7 hours | 194/713 | 1.00 (ref) |  | 1.00 (ref) |  | 1.00 (ref) |  |
| 8 hours | 121/353 | 1.36 (1.08, 1.72) | 0.010 | 1.41 (1.12, 1.78) | 0.004 | 1.43 (1.13, 1.81) | 0.003 |
| ≥9 hours | 9/43 | 0.69 (0.34, 1.37) | 0.285 | 0.67 (0.33, 1.35) | 0.257 | 0.70 (0.35, 1.38) | 0.301 |

Abbreviations: CI, confidence intervals; HR, hazard ratio; na, not applicable (N cases≤5); ref, reference; SD, standard deviation.

^a^ Adjusted for age (time-scale), sex, ethnicity, education, occupational position, and marital status.

^b^ Additionally adjusted for alcohol consumption, physical activity, smoking status, fruit and vegetable consumption, BMI, hypertension, and use of sleep medication.

^c^ Chronic disease among diabetes, cancer, coronary heart disease, stroke, heart failure, chronic obstructive pulmonary disease, chronic kidney disease, liver disease, depression, dementia, other mental disorder, Parkinson’s disease, and arthritis/rheumatoid arthritis; Multimorbidity defined as 2 or more of these diseases.
