# Supplementary material for: Association of sleep duration at age 50, 60, and 70 years with risk of multimorbidity in the UK: 25-year follow-up of the Whitehall II cohort study
Source: PLoS Med. 2022 Oct 18;19(10):e1004109. doi: 10.1371/journal.pmed.1004109 (PMC9578599; doi:10.1371/journal.pmed.1004109)
Supplement: Attachment — Submitted filename: response-final.docx [file pmed.1004109.s018.docx]

**Editor**

**PLOS Medicine**

26^th^ July 2022

Dear Dr. Moyer,

**RE: Manuscript ID PMEDICINE-D-22-00972R1 entitled "Association of sleep duration at age 50, 60, and 70 with risk of multimorbidity: 25-year follow-up of the Whitehall II cohort study"**

Thank you for your comments and those from the reviewers on our manuscript. We are delighted to be invited to revise and resubmit the manuscript. Please see below our detailed response to the comments from the editorial board and the four reviewers. We hope that the revised version of our paper has addressed all concerns and is now suitable for publication.

We would like to thank you for considering our paper and look forward to hearing from you.

Yours sincerely,

Séverine Sabia, on behalf of all authors.

Université Paris Cité, Inserm 1153

EpiAgeing “Epidemiology of Ageing & Neurodegenerative diseases”

10 avenue de Verdun

75010 Paris, France

Tel : +33 (0)1 57 27 90 46

Email: [severine.sabia@inserm.fr](mailto:severine.sabia@inserm.fr)

**EDITORS’ COMMENTS**

**1.** Title: Please revise your title according to PLOS Medicine's style. Your title must be nondeclarative and not a question. It should begin with main concept if possible. "Effect of" should be used only if causality can be inferred, i.e., for an RCT. Please place the study design ("A randomized controlled trial," "A retrospective study," "A modelling study," etc.) in the subtitle (ie, after a colon).

**OUR RESPONSE:** Thank you. We have changed the title as follows (page 1, lines 1-2 from the clean version of the revised manuscript):

*“Association of sleep duration at age 50, 60, and 70 with risk of multimorbidity: 25-year follow-up of the Whitehall II cohort study”.*

**2.** Data availability statement: The Data Availability Statement (DAS) requires revision. For each data source used in your study:

a) If the data are freely or publicly available, note this and state the location of the data: within the paper, in Supporting Information files, or in a public repository (include the DOI or accession number).

b) If the data are owned by a third party but freely available upon request, please note this and state the owner of the data set and contact information for data requests (web or email address). Note that a study author cannot be the contact person for the data.

c) If the data are not freely available, please describe briefly the ethical, legal, or contractual restriction that prevents you from sharing it. Please also include an appropriate contact (web or email address) for inquiries (again, this cannot be a study author).

**OUR RESPONSE:** The Data Availability Statement has been revised as: *“Data cannot be made publicly available because of ethics and IRB restrictions. However, the data are available to bona fine researchers at small fee - to cover data management costs - via a data sharing portal allowing access to undertake analyses within a secure portal,* [*https://portal.dementiasplatform.uk/*](https://portal.dementiasplatform.uk/)*”.*

**3.** Line numbers: Please provide line numbers, running continuously throughout the document, with the revised version.

**OUR RESPONSE:** Line numbers have been added to the revised manuscript as requested.

**4.** Abstract: Methods and Findings: Please provide some summary information on the Whitehall II population. Please mention the study dates, and the setting of your study. Please mention the study design.

**OUR RESPONSE:** We have now added summary demographic information on participants as follows (page 2, lines 28-32 and 37):

***“****Data were drawn from the prospective Whitehall II cohort study, established in 1985-1988 on 10,308 persons employed in the London offices of the British civil service. Self-reported sleep duration was measured six times between 1985-1988 and 2015-2016 and data on sleep duration was extracted at age 50 (mean age (standard deviation)=50.6 (2.6) years), 60 (60.3 (2.2) years) and 70 (69.2 (1.9) years). Incidence of multimorbidity was defined as having two or more of 13 chronic diseases, follow-up up to March 2019.”*

*“A total of 7,864 (32.5% women) participants free of multimorbidity had data on sleep duration at age 50…”*

**5.** Abstract: Methods and Findings: Please quantify the main results with both 95% CIs and p values. Please ensure that all numbers presented in the abstract are present and identical to numbers presented in the main manuscript text.

**OUR RESPONSE:** Thank you, we have revised the abstract as requested (pages 2-3).

**6.** Abstract: Methods and Findings: In the last sentence of the Abstract Methods and Findings section, please describe the main limitation(s) of the study's methodology.

**OUR RESPONSE:** The following sentence has been added as the end of the Abstract Methods and Findings section (page 3, lines 49-50):

*“The main limitation of this study is the small number of cases in this long sleep category, not allowing to draw firm conclusions to be drawn for this category.”*

**7.** Abstract: Conclusions: Please address the study implications without overreaching what can be concluded from the data; the phrase "In this study, we observed ..." may be useful. Please revise the Conclusions section such that it provides an interpretation of the study based on the results presented in the abstract, emphasizing what is new without overstating your conclusions. For example, the study does not seem to report evidence suggesting that long sleep is a marker of underlying health conditions.

**OUR RESPONSE:** Thank you. The conclusions of the Abstract have been revised as follows (page 3, lines 52-55):

*“In this study, we observed short sleep duration to be associated with risk of chronic disease and subsequent multimorbidity but not with progression to death.* *There was no robust evidence of an increased risk of chronic disease among those with long sleep duration at age. Our findings suggest a role of short sleep duration in the risk of multimorbidity.”*

**8.** Methods: How was ethnicity defined and by whom?

**OUR RESPONSE:** Information on the measure of ethnicity has been provided as follows (page 10, lines 196-198):

*“**Socio-demographic factors included age, sex, ethnicity (response to a question using the categories “White”, “South Asian”, “Black”, and “Other” and categorized in the analysis as White and non-White, due to the small numbers in the latter group),…”*

**9.** Methods: Repeated measures design: Please describe how clustering of repeat measurements at the individual-level was accounted for.

**OUR RESPONSE:** Apologies for the confusion. Measures of sleep duration over the course of the study were used to extract sleep duration at age 50, 60, and 70. Then the analyses on sleep duration and multimorbidity was examined in separate analyses for measures of sleep duration at age 50, 60, and 70. This has been clarified as follows (page 11, lines 216-222):

*“**The association of sleep duration at age 50, 60, and 70 with incident multimorbidity was examined in separated models. The analyses were undertaken using Cox proportional-hazards regression with age as the time scale in participants free from multimorbidity at the measurement of sleep duration. Data were censored at date of multimorbidity diagnosis, death to account for competing risk [37], or 31^st^ of March 2019, whichever came first. In analysis of sleep duration at age 50, age at the beginning of the follow-up was the age at clinical assessment closest to 50 years from which the sleep duration measure and covariates were drawn. A similar approach was used in analyses of sleep duration at age 60 and 70.”*

In response to comment 4 from Reviewer 4, we added a post-hoc analysis on trajectories of sleep duration between age 50 and 70 and incident multimorbidity. Results showing persistent short sleep duration to be associated with increased risk of multimorbidity are reported in the Results section (page 19, lines 348-356) and S9 Table.

**10.** Methods: “We repeated this analysis using inverse probability weighting to account for missing data…” Please provide more information/a table describing the nature and amount of missing data.

**OUR RESPONSE:** The sample selection for the analysis of sleep duration after one chronic disease and risk of subsequent multimorbidity and mortality is shown in S1 Fig and missing data pattern now described in the Results section as follows (page 22, lines 397-400):

*“**A total of 6,546 participants were diagnosed with one of the 13 chronic diseases considered during the follow-up period, constituting the target population of this analysis. Among them, 2,442 did not have data on sleep duration after the onset of this first chronic disease onset, 464 had data but only after the onset of multimorbidity, and 42 had missing covariates, leading to an analytical sample of 3,702 participants (S1 Fig).”*

**11.** Methods: “...(3) by the association between accelerometer-assessed sleep duration at mean age 69 years and incident multimorbidity. The accelerometer measure and covariates were drawn from the 2012-2013 wave of data collection. Given the detailed data on sleep duration extracted from the accelerometer, we used restricted cubic spline regressions with Harrell knots [32], Stata command xblc [33], with 7-hour sleep as the reference to examine the shape of the association between sleep duration and dementia risk.” Please clarify if the accelerometer data were available for participants at other ages, as well. Please clarify the reason why the association between sleep duration and dementia is highlighted in this analysis.

**OUR RESPONSE:** Thank you for this comment. Information on age at the accelerometer measure (age range: 60 to 83 years) has been added to the Methods section (page 11, line 229). The accelerometer measure was a one-off addition to the main data collection, so we do not have repeat accelerometer data. This information has been added in the Methods section describing the measure of sleep duration (page 8, lines 142-150). Apologies for the typo re dementia in the spline regression, now corrected as “multimorbidity risk”.

**12.** Methods: Please ensure that the study is reported according to the STROBE guideline, and include the completed STROBE checklist as Supporting Information. Please add the following statement, or similar, to the Methods: "This study is reported as per the Strengthening the Reporting of Observational Studies in Epidemiology (STROBE) guideline (S1 Checklist)."

The STROBE guideline can be found here: <http://www.equator-network.org/reporting-guidelines/strobe/>

When completing the checklist, please use section and paragraph numbers, rather than page numbers.

**OUR RESPONSE:** The STROBE checklist has been added as Supporting information as now stated at the start of the Methods section (page 7, lines 115-116):

*“This study is reported following the Strengthening the Reporting of Observational Studies in Epidemiology (STROBE) guideline (S1 Checklist).”*

**13.** Methods: Did your study have a prospective protocol or analysis plan? Please state this (either way) early in the Methods section.

a) If a prospective analysis plan (from your funding proposal, IRB or other ethics committee submission, study protocol, or other planning document written before analyzing the data) was used in designing the study, please include the relevant prospectively written document with your revised manuscript as a Supporting Information file to be published alongside your study, and cite it in the Methods section. A legend for this file should be included at the end of your manuscript.

b) If no such document exists, please make sure that the Methods section transparently describes when analyses were planned, and when/why any data-driven changes to analyses took place.

c) In either case, changes in the analysis-- including those made in response to peer review comments-- should be identified as such in the Methods section of the paper, with rationale.

**OUR RESPONSE:** The analysis plan is now provided in Supporting information file (S1 Text) and the new analyses undertaken to address comments from reviewers is referred to as “post-hoc” analyses (first sentence of the Statistical analysis (page 11, lines 213-214)):

*“The analysis plan was developed prior to data analysis (S1 Text). The analyses referred to as post hoc analyses were in response to suggestions from reviewers.”*

**14.** Results: Please provide both 95% CIs and p values for all results presented in the text.

**OUR RESPONSE:** The results section has been revised accordingly.

**15.** Results: “Sleep duration ≥9 hours at age 60 (1.57, 1.17-2.09) and 70 (1.45, 1.05-1.98) years was associated with higher risk of multimorbidity” Please also present the result for sleep duration greater than 9 hours at age 50.

**OUR RESPONSE:** The information is now provided as follows (page 16, lines 302-305)*:*

*“**the association of sleep duration ≥9 hours at age 50 with incident multimorbidity did not reach statistical significance (HR=1.39, 0.98-1.96; p=0.067), while sleep duration ≥9 hours at age 60 (HR=1.54, 1.15-2.06; p=0.003) and 70 (HR=1.51, 1.10-2.08; p=0.010) was associated with higher risk of multimorbidity.”*

**16.** Results: “the pattern of associations was similar for analyses using sleep duration at age 50, 60, and 70 years although some of the associations were under-powered.” We suggest providing more detail on how the analyses were determined to be under powered, or changing the wording to reflect that while the results of these analyses were directionally similar, they were no longer statistically significant.

**OUR RESPONSE:** Thank you we have rephrased the sentence as requested (page 18, lines 315-318): *“the pattern of associations was similar to the main analyses for sleep duration at age 50, 60, and 70 years apart from the association with sleep duration ≥9 hours at age 70 that did not reach significance although the effect size was comparable (HR=1.52, 0.98-2.35; p=0.063).”*

**17.** Results: “Accelerometer data and covariates were available on 3920 participants.” Please clarify the age group for this analysis. Please provide the HR, 95% CI and p values for this analysis.

**OUR RESPONSE:** Information on age has been added to the text in the Results section (page 18, line 320) and in the title of Figure 3. We have also added the frequency distribution of accelerometer-assessed sleep duration in panel D of Figure 3. As shown in this Figure, the number of incident multimorbidity cases below 6 hours of sleep and above 8 hours was small, so we chose to use the accelerometer data to examine the shape of the association rather than formal estimates of association. We have added this explanation to the Results section (page 18, lines 326-328).

**18.** Results: For example at: “Sleep duration ≥9 hours was also associated with a higher risk of multimorbidity (1.46, 1.07-1.99), although the association was attenuated after adjustment for behavioral and health-related factors (1.36, 1.00-1.86).” Throughout the results section, it is not always clear where the unadjusted results, or results from Model 1 or Model 2 are being presented. Please indicate which factors are adjusted for each analysis, unless this is consistent throughout.

**OUR RESPONSE:** Apologies, we have revised the manuscript to address this concern.

**19.** Discussion: Please present and organize the Discussion as follows: a short, clear summary of the article's findings; what the study adds to existing research and where and why the results may differ from previous research; strengths and limitations of the study; implications and next steps for research, clinical practice, and/or public policy; one-paragraph conclusion.

**OUR RESPONSE:** Thank you, the discussion has been revised accordingly.

**20.** Discussion: Here and throughout, please avoid any language that implies causality: “The analyses of health state transitions also indicated that long sleep at age 50 does not affect disease progression…”

**OUR RESPONSE:** We have revised the discussion section to avoid language that could imply causality.

**21.** Discussion: There was no evidence in the present data that short sleep duration plays an important role in disease prognosis…” Please clarify that you are referring to mortality.

**OUR RESPONSE:** The sentence has been revised as follows (page 25, lines 452-453):

*“**There was no evidence in the present data that short sleep duration was associated with progression to death among those with existing chronic disease(s).”*

**22.** Figures and Tables: Please be sure that there are titles and legends for each individual table and figure, including those in the Supporting Information. Please fully define any abbreviations used in figures and tables within the text of the legend.

**OUR RESPONSE:** We have verified all titles and legends to ensure all required information is provided.

**23.** Table 1 (and eTable 1 and eTable 2): Please report on the full set of characteristics of the study population (e.g. where there are more than two categories for each variable). Please provide a summary for the total group, in addition to the breakdown by sleep duration.

**OUR RESPONSE:** Thank you. We have now added in each descriptive table a column for the full population as well as lines for each group of the categorical variables.

**24.** Table 2, Table 3, Table 4 and eTable 4, eTable 5, and eTable 6: Please report exact p values, instead of *p<0.05. Please also present the results from unadjusted analyses.

**OUR RESPONSE:** Tables have been revised accordingly.

**Reviewer 1**

This study examines sleep duration as a potential risk factor for first chronic disease, subsequent multimorbidity and mortality using data spanning 25 years.

Comments:

**1.** "Self-reported sleep duration was measured at age 50 and incidence of multimorbidity was defined as presence of two or more of 13 chronic diseases."

Can the authors please comment on the potential for self reporting bias?

**OUR RESPONSE:** The following text has been added to the discussion to comment on the self-reported nature of sleep duration (page 26, lines 478-481 of the clean version of the revised manuscript):

*“First, like most large scale studies on sleep we used self-reported sleep which is likely to be subject to reporting bias. Although the correlation between accelerometer-assessed and self-reported sleep duration was moderate in our study the shape of the association with multimorbidity risk was similar with both measures.”*

**2.** "In 2012-2013 an accelerometer sub-study was undertaken on participants who attended the central London research clinic or were assessed at home if they resided in the South-Eastern regions of England. Wrist-worn accelerometers, the GENEActiv (Activinsights Ltd, Kimbolton, UK), were worn 24h over 9 consecutive days [22]. Sleep duration was estimated using a validated algorithm guided by a sleep log [23]; data from the first and last nights were removed leading to data over 7 nights. Usual daily sleep duration was estimated as the mean of sleep duration over the 7 nights and for those with less than 7 7 nights of measurement, weighted average of sleep duration was calculated as: 5 x week night sleep duration + 2 x weekend night sleep duration)/7. "

Are the authors able to explore and comment on how this accelerometer data compares to self-reported estimates of sleep duration?

**OUR RESPONSE:** Thank you, please see response to the comment above; in response the following text that has been added to the Results section (page 18, lines 319-325):

*“**Accelerometer data and covariates were available on 3,920 participants who took part to the accelerometer sub-study in 2012-2013. Of them, 3,368 participants (mean age (SD)=68.9 (5.6), range=60-83 years) were free of multimorbidity and were included in the analysis. During a mean follow-up of 6 years, 601 developed multimorbidity. The correlation between accelerometer- and questionnaire-assessed sleep duration was moderate (Pearson correlation=0.41, p<0.001). The shape of the association between accelerometer-assessed sleep duration and incident multimorbidity was similar to that observed with self-reported sleep duration, with the lowest risk of incident multimorbidity seen at 7 hours of sleep (Fig 3).”*

**3.** "The association of sleep duration at age 50, 60, and 70 with risk of incident multimorbidity was examined using Cox proportional-hazards regression with age as the time scale in participants free from multimorbidity at the measurement of sleep duration. Data were censored at date of multimorbidity diagnosis, death to account for competing risk [30, 31], or 31st of March 2019, whichever came first. Age at the beginning of the follow-up was the age at clinical assessment closest to target age in the analyses (50, 60, and 70 years) from which the sleep duration measure and covariates were drawn. The proportional hazards assumption was verified using Schoenfeld residuals. Analyses were adjusted first for sociodemographic measures (Model 1), and then additionally for behavioral and health-related factors (Model 2)."

The authors have applied rigorous modelling methods, appropriately adjusting for potential confounding and reporting the results accurately.

**OUR RESPONSE:** Thank you for this positive feedback.

**4.** "To examine the robustness of our findings, we repeated the main analysis (1) in participants free from any chronic disease at the sleep measurement, (2) excluding users of sleep medication and (3) by investigating the association between accelerometer-assessed sleep duration at mean age 69 years and incident multimorbidity."

and

"In additional analyses, we examined the association of sleep duration after onset of a first chronic disease with transitions to multimorbidity and death, again using a multi-state model. The follow-up here started at the measure of first sleep duration following the onset of a first chronic disease. We repeated this analysis using inverse probability weighting to account for missing data that arise from the fact that 10 sleep duration was not measured after a first chronic disease and before onset of multimorbidity in some participants [34]"

The authors have suitably conducted additional analyses which help to demonstrate the robustness of the study findings.

**OUR RESPONSE:** Thank you to point to the strengths of our analysis.

**5.** "No association was found for mortality among those with existing chronic diseases. "

Did the authors consider exploring an overall transition analysis with death modelled as the outcome for the whole cohort (i.e. not conditional on the non-existence or existence of chronic disease(s))?

**OUR RESPONSE:** Thank you for this excellent suggestion. In order to provide a comprehensive picture of the association of sleep duration at age 50 with risk of mortality, we added a supplementary table (S13 Table) showing the association of sleep duration at age 50 and incident mortality irrespective of the presence of chronic disease(s). Findings are reported in the Results section as follows (page 20, lines 377-381):

*“**In post-hoc analysis, we also examined the association between sleep duration at age 50 and risk of mortality without considering chronic diseases over the follow-up; in analysis adjusted for sociodemographic, behavioral and health-related factors, sleep duration ≤5 hours (HR=1.25, 1.02-1.53; p=0.034) and of 6 hours (HR=1.17, 1.04-1.32; p=0.008) was associated with higher risk of mortality over a mean follow-up of 25.2 (SD=6.9) years (S13 Table).”*

**6.** "In analyses excluding participants with any of the 13 chronic diseases at measurement of sleep duration (eTable 4), the pattern of associations was similar for analyses using sleep duration at age 50, 60, and 70 years although some of the associations were under-powered."

Can the authors please further expand on the power analysis they refer to here?

**OUR RESPONSE:** We have removed the term under-powered and revised the sentence as follows (page 18, lines 314-317):

*“**In sensitivity analyses excluding participants with any of the 13 chronic diseases at the measure of sleep duration (S4 Table), the pattern of associations was similar to the main analyses for sleep duration at age 50, 60, and 70 years apart from the association with sleep duration ≥9 hours at age 70 that did not reach significance although the effect size was comparable (HR=1.52, 0.98-2.35; p=0.063).”*

**7.** "The shape of the association between accelerometer-assessed sleep duration and incident multimorbidity confirmed that 7 hours of sleep was associated with the lowest risk of incident multimorbidity (Figure 3). "

Can the authors please comment on Figure 3b, which shows adjusted CIs crossing zero, suggesting no evidence of an increased HR for < or > 7 hours sleep?

**OUR RESPONSE:** Thank you for this comment. Unfortunately, the number of multimorbidity cases in the non-reference groups is too small to allow estimation of HR and 95% CIs. The figure is primarily used to show the shape of the association; we have added the following text to clarify the focus of this analysis in the Results section (page 18, lines 323-328):

*“**The shape of the association between accelerometer-assessed sleep duration and incident multimorbidity was similar to that observed with self-reported sleep duration, with the lowest risk of incident multimorbidity seen at 7 hours of sleep (Fig 3). Given the small number of incident multimorbidity cases in participants in less than 6 hours and more than 8 hours categories (Fig 3, panel D), the focus here was on the shape of the association rather than estimates of the associations.”*

**8.** Table 1 (and eTables 1 and 2): Can the authors consider additionally presenting BMI as a continuous variable here please?

**OUR RESPONSE:** As requested, BMI is now also presented as a continuous variable in Table 1, and in S1 & S2 Tables.

**Reviewer 2 comments**

This is a prospective cohort study addressing the role of sleep duration in the progression from a disease-free state to chronic disease, multimorbidity, and death shows short sleep duration to be a risk factor for the onset of chronic disease and multimorbidity but not for subsequent mortality in those with chronic disease(s). The paper is well written and with an extensive and detailed statistical analysis. I would like to point only one minor suggestion, please see the details bellow.

**OUR RESPONSE:** We thank the reviewer for this positive feedback.

Page 9 - Statistical analysis, section Association between sleep duration at different ages and incident multimorbidity, last line "…with 7-hour sleep as the reference to examine the shape of the association between sleep duration and dementia risk."

This sentence does not seem to make sense here at this paper since assessing dementia risk was not an objective of this paper. Dementia was only assessed among the remain chronic diseases.

**OUR RESPONSE:** Apologies for the typo, a left-over from the title of a figure for another paper. We corrected the text as: "…with 7-hour sleep as the reference to explore the shape of the association between sleep duration and multimorbidity risk."

**Reviewer 3 comments**

This 25-year population-based cohort study leverages data on 13 chronic diseases to assess the relation between (self-reported) sleep duration and multimorbidity at different ages. Using sophisticated multistate models, they are able to show that short sleep duration (shorter or equal to 5 hours) increases the relative risk of any chronic disease and multimorbidity with 20% compared to longer sleep duration, but not to death.

The White Hall II study has several key strengths, including long-term, meticulously acquired health data on thousands of individuals. It also repeatedly assessed sleep behavior at various ages, and what I particularly like: a simultaneous assessment of objective sleep duration with that of self-report, allowing verification of self-reported data, as well as the authors' endeavor to repeat analyses with inverse probability weighting. Finally, I would like to congratulate the authors with their careful selection of diseases to define multimorbidity, while rightfully excluding risk factors.

**OUR RESPONSE:** Thank you for this summary of the strengths of the study.

I have three main concerns, and several minor suggestions to further improve the paper.

**1.** I like the analyses and idea of this study, but I struggle with interpreting the relevance of findings for public health or clinicians. Assessing relations with general health markers such as physical activity, sleep or diet with multimorbidity seems troublesome for etiological purposes since it is hard to say what specific disease (combinations) particularly drive associations. As this study appears to be of etiological origin (presentation of relative risks, and "Our findings support an etiological role of short sleep duration for onset of chronic diseases, including a first chronic disease and subsequent multimorbidity"), it seems that we would like to know how short sleep duration pathophysiologically relates to what specific diseases. This provides clinicians a direct target for intervention.

**OUR RESPONSE:** Thank you for this interesting comment. In response to comment 20 from the editors we have removed references to an etiological interpretation. From the populations sciences perspective our focus is on “whether” sleep duration is associated with multimorbidity rather than “how” it is associated. Multimorbidity is increasingly the norm rather than the exception at older ages with calls to adapt the health care system, highlighted in previous reports (references 6 and 42 in the manuscript).

**2.** Only short sleep duration (equal or less than 5 hours) related to disease and multimorbidity. It raises the question why particularly this cutoff was chosen, as 5 hours is exceptionally short for an average weekday, and indeed prevalence is low (7.5%). It would rather be interesting to study the relationship continuously, by entering sleep duration per 1 hour into the models.

**OUR RESPONSE:** Both short and long sleep durations have been shown previously to be associated with higher risk of incident chronic disease (reference 1-3) and the biological pathways linking short and long sleep duration with health might be different (references 15, 16, 19). We used 5 categories of sleep duration (≤5 hours, 6 hours, 7 hours, 8 hours, ≥9 hours) as is the convention to be able to capture any potential higher risk at both extremes of the distribution. Entering sleep duration as a continuous variable would not fit the U-shaped association, as shown in the accelerometer-measure of sleep duration (Fig 3).

**3.** The authors use sophisticated statistical models to disentangle relations over the life course. It is perhaps valuable to explain to the (general) reader why these models are relevant for this particular study compared to for instance traditional cox models. This can be done briefly in the introduction, and more elaborately in the methods part.

**OUR RESPONSE:** Thank you for the suggestion, we have added the following text to the manuscript:

Introduction (page 6, lines 102-105 of the clean version of the revised manuscript):

*“A second objective was to determine whether sleep duration at age 50 shapes the natural course of chronic disease, from a healthy state, to a first chronic disease, multimorbidity, and death using multi-state models to examine the association of sleep duration at age 50 with transitions between each of these health states.”*

Methods (page 12, lines 244-250):

*“**Among participants at age 50, free from the 13 chronic diseases considered here, multi-state models were used (Fig 1) to determine the association of sleep duration at age 50 with transitions from: A) a healthy state to a first chronic disease (any from the list of 13 diseases considered); B) a healthy state to death (in those who remained free from any of the 13 diseases during follow-up); C) a first chronic disease to multimorbidity; D) a first chronic disease to death; and E) multimorbidity to death, with follow-up starting at age 50. The advantage of multi-state models is that they take into account the time spent within each health state to estimate probabilities of transitions between each state.”*

Abstract

**4.** Rather the increased risk of multimorbidity associated with long sleep duration in those with existing disease might reflect the need for longer sleep in those with underlying chronic conditions. This is an interesting interpretation, but it makes me wonder why this should not also hold for the relation with death?

**OUR RESPONSE:** We agree with the reviewer’s interpretation, the hypothesis might also hold for the association between long sleep and mortality. We chose not to comment as we did not find an association between long sleep and higher risk of mortality in our study, probably due to small number of cases in this group. However, following revision on the abstract, the conclusion has been modified and it no longer includes this sentence. It now reads (page 3, lines 53-54): “There was no robust evidence of an increased risk of chronic disease among those with long sleep duration at age 50.”

**5.** briefly add some demographics of the study population, including prevalence of short sleep duration as well as limitations of the study

**OUR RESPONSE:** Thank you, we have revised the abstract to include this information (page 2).

Introduction

**6.** although a number of outstanding questions remain regarding the nature of this relation. I would then rephrase "one, multiple.." to "First, multiple.." to increase readability.

**OUR RESPONSE:** We have made the changes as suggested (page 6).

Methods:

**7.** Through linkage with medical records, data on a host of diseases are available. Were diagnoses also verified by study physicians? Is there any data available on the accuracy of these records?

**OUR RESPONSE:** Identification of chronic diseases using linkage to medical records has been validated for multiple outcomes, such as cardiovascular disease, dementia, and mental disorders. References for these validation studies have been added to the Methods section (page 8, lines 174, 175, 181, and 184; references 34 to 36 in the manuscript). Use of electronic health records for chronic diseases is common in the UK due to the nature of the National Health Service (NHS), the provider of healthcare for UK residents. Large scale studies such as the UK Biobank cohort also use this resource (Gao et al, Age Ageing 2022) because compared to self-reports or study based diagnosis, data on chronic conditions are available on all participants whether or not they continue to participate in the study over time. This approach is likely to reduce the effects of selection bias.

Reference:

Gao X et al. Mortality and morbidity risk prediction for older former smokers based on a score of smoking history: evidence from UK Biobank and ESTHER cohorts. Age and Ageing. 2022;51(7):afac154. <https://doi.org/10.1093/ageing/afac154>

**8.** Apart from inverse probability weighting, how did the authors handle missing data in the main analyses?

**OUR RESPONSE:** In the original submission, we conducted sensitivity analysis using inverse probability weighting for the analysis on sleep duration after a first chronic disease and incident multimorbidity as non-participation was considerable in this group. Following the reviewer’s comment, we have now added post-hoc analyses repeating all main analyses using inverse probability weighting to account for missing data. Findings were in accordance with the main analyses (S6 and S12 Tables) as now reported in the Results section (page 19, lines 345-346; page 20, lines 376-377).

**9.** Perhaps add STROBE checklist

**OUR RESPONSE:** As also suggested by the editor, we have now added a STROBE checklist (S1 Checklist).

**10.** add analysis with E-value, to address potential residual confounding that could not be adjusted for

**OUR RESPONSE:** Given the range of analyses undertaken, we chose not to calculate E-values for every estimates as they are not widely used in the literature due to difficulties in interpretation. For example, in Table 2, a putative confounder would need to have an association that has a risk ratio of 2 to explain away the association between sleep duration ≤5 hours at age 50 and incident multimorbidity. We have added the following text to the discussion to account for the reviewer’s comment (page 26, lines 487-490):

*“**despite the use of several covariates residual confounding can be an issue in observational studies.*

*For example, considering the wide range of covariates a confounder would need to have a risk ratio of 2 (E-value) with both the exposure and the outcome to explain away the association between sleep duration at age 50 and multimorbidity.”*

Discussion

**11.** Is there any data available on sleep quality? This seems relevant to include as authors specifically relate to "Long sleep duration might reflect poor overall sleep quality that could have a detrimental impact on health".

**OUR RESPONSE**: Thank you for the suggestion, we have now added post-hoc analysis using data on sleep quality. These data were available at age 60 and 70 (not age 50). We found short but not long sleep duration to be associated with greater sleep disturbance and that sleep disturbance was associated with incident multimorbidity. The following text has been added to the manuscript:

Methods (page 8, lines 151-159):

***“****In post-hoc analysis, we used data on sleep quality measured using the Jenkins sleep problems scale [27]. This measure was introduced to the study questionnaire in 1997-1999 and repeated at following study waves, allowing us to extract data on sleep quality at age 60 and 70, but not age 50. Participants were asked how often in the past month they had experienced the following symptoms: (1) trouble falling asleep, (2) waking up several times per night, (3) trouble staying asleep (including waking far too early), and (4) disturbed or restless sleep. The following response categories were available: Not at all (scored 0), 1-3 days (scored 1), 4-7 days (scored 2), 8-14 days (scored 3), 15-21 days (scored 4) and 22-31 days (scored 5). The sum of these items was then used as a continuous scale to measure sleep problems. The score was further dichotomized to reflect low sleep disturbance (0-11) and high sleep disturbance (12-20) [28].****”***

Results (page 19, lines 357-364):

*“**In a fourth post-hoc analysis, sleep disturbances at age 60 and 70, measured using the Jenkins sleep problems scale, were examined and found to be greater in those with sleep duration ≤5 hours at age 60 as compared to with 7 hours of sleep (mean (SD) Jenkins sleep problems score = 10.1 (SD=5.7) vs 4.4 (SD=3.5), p<0.001; S10 Table) but not in those with sleep duration ≥9 hours (4.4 (SD=4.1) vs 4.4 (3.5); p=0.925). A similar pattern was observed at age 70 (S10 Table). Greater sleep disturbance at age 60 and at age 70 (HR=1.03, 1.02-1.04 per 1-point increase in the score at both ages; p<0.001) was associated with increased risk of multimorbidity in analyses adjusted for sociodemographic, behavioral, and health-related factors (S11 Table).”*

Discussion:

*“**Short sleep duration was also associated with greater sleep disturbances – itself associated with increased risk of multimorbidity in the present study as well as in a previous study [31] – suggesting that short sleep duration might be a marker of poor sleep quality.”* (page 25, lines 448-450)

*“**Long sleep duration has been hypothesized to reflect poor overall sleep quality that could have a detrimental impact on health [15, 19, 31], although this hypothesis was not supported in our study where the sleep disturbances were similar in those sleeping ≥9 hours and those sleeping 7 hours.”* (page 25, lines 458-461)

Limitations of study are mentioned but could be expanded:

**12.** if data on sleep quality is not present, this should be added

**OUR RESPONSE:** Please see response to the previous comment. We have added the following text to highlight the absence of sleep quality data at age 50 (page 26, line 482):

*“**data on sleep quality were available only at age 60 and 70.”*

**13.** perhaps the authors can reflect on the diversity of their study in terms of ethnicity?

**OUR RESPONSE:** Thank you, we have added the following text to the limitations section (page 26, line 485-487):

*“**Fourth, participants were mostly of white ethnicity, reflecting the population of the country in 1991 [41], and whether results are generalizable to other populations is unknown.”*

**14.** Since duration does not directly tell us something about quality, I am not sure whether I agree with the following statement: "Our findings suggest short sleep duration in midlife and old age is associated with higher risk of onset of a first disease, and subsequent multimorbidity among those with an existing condition. This supports promotion of good sleep hygiene in both primary and secondary prevention." Can the authors rephrase? What should clinicians do based on these findings, sole advice to sleep longer or stay longer in bed seems not so relevant if patients are not able to sleep.

**OUR RESPONSE:** As reported in response to comment 11, we have added analyses on sleep quality showing its association at age 60 and 70 with incident multimorbidity. We have revised the conclusion as follows (page 27, lines 503-506):

*“In conclusions, findings from the present study suggest short sleep duration in midlife and old age is associated with higher risk of onset of chronic disease and multimorbidity. These findings support the promotion of good sleep hygiene in both primary and secondary prevention by targeting behavioral and environmental conditions that affect sleep duration and quality [44].”*

**Reviewer 4 comments**

**1.** The present study analyses the association between sleep duration and risk of first chronic disease, multimorbidity and death. While the analytical approach seems robust, there are several aspects that may threaten the internal validity of the findings.

**OUR RESPONSE:** Thank you. Please see our point-by-point response to the reviewer’s comment.

**2.** The main problem I see with this study is the low number of chronic diseases taken into consideration. This increases the risk of reverse causality given the potential heterogeneity in subclinical health states leading to and departing from a state of multimorbidity. If the authors cannot account for more diseases due to data availability issues, they should consider adjusting their models for some other comprehensive measure of health, e.g., walking speed, number of medications, muscle strength, etc.

**OUR RESPONSE:** For now a gold standard definition of multimorbidity does not exist. Our list was defined using the literature in this domain and the proviso that at least 12 conditions be used to define multimorbidity (Nicholson et al, Health Psychol, 2019) and that they reflect dysfunction in multiple organs (Ho et al, Lancet Public Health, 2021). Too large a list of conditions makes the concept meaningless for clinicians and for identifying prevention targets. For example, a recent paper on the multimorbidity frailty index included 38 conditions, including for example cough, dizziness and giddiness, as well as cerebrovascular disease (Lai et al, Arch Gerontol Geriatr, May-Jun 2022), leading to more than 70% of older adults being multimorbid. We chose to concentrate on 13 chronic diseases that cover a large set of body functions and have been previously used in the literature (Ho et al, Lancet Public Health, 2021; Ben-Hassen, BMJ, 2022).

References:

- Nicholson et al. The measurement of multimorbidity. Health Psychol. 2019;38(9):783-90. Epub 2019/04/26. doi: 10.1037/hea0000739. PubMed PMID: 31021126. (reference 29 in the manuscript)
- Ho et al. Examining variation in the measurement of multimorbidity in research: a systematic review of 566 studies. Lancet Public Health. 2021;6(8):e587-e97. Epub 20210622. doi: 10.1016/S2468-2667(21)00107-9. PubMed PMID: 34166630. (reference 30 in the manuscript)
- Lai et al. Development of frailty index using ICD-10 codes to predict mortality and rehospitalisation of older adults: An update fo the multimorbidity frailty index. Arch Greontol Geriatr. 2022; 100:104646. doi: 10.1016/j.archger.2022.104646 PMID: 35183986
- Ben Hassen et al. Association between age at onset of multimorbidity and incidence of dementia: 30 year follow-up in Whitehall II prospective cohort study. BMJ. 2022;376:e068005. Epub 2022/02/04. doi: 10.1136/bmj-2021-068005. PubMed PMID: 35110302. (reference 33 in the manuscript)

**3.** One still wonders whether the potential impact of sleep duration on incident chronic disease and multimorbidity would still exist above and beyond the link with specific chronic conditions. One possible way to verify this is that the authors repeat the analyses after removing each of the 13 chronic conditions from their count, one at a time.

**OUR RESPONSE:** Thank you for this excellent suggestion. In order to examine the role of each chronic disease in the association between sleep duration and multimorbidity we undertook further analyses. Findings are reported in S7 Table and results commented in the Results section as follows (page 19, lines 346-348 of the clean version of the manuscript):

*“**analysis excluding one disease at a time from the definition of multimorbidity showed results to be robust to the list of chronic diseases used to define multimorbidity (S7 Table).”*

**4.** Simply measuring sleep duration in absolute terms seems suboptimal. Data on sleep quality (e.g., sleep efficiency, sleep latency) have been shown to be important aspects of sleep. Moreover, assessing sleep duration in absolute terms could result in bias for those people with usual shorter/longer sleep duration. Also, what is the stability of self-reported sleep duration across time? Given that the authors have access to several of these measures over time, it may be pertinent to check this; even more so considering the long follow-up time for the 50-year and 60-year groups (22.6 and 13.4 years respectively).

**OUR RESPONSE:** These are important considerations. We have added analyses on sleep quality in post-hoc analysis (S10 and S11 Tables), please see our response to comment 11 from reviewer 3 for details on this analysis. Regarding stability of sleep duration over time, we thank the reviewer for their suggestion. We have added data on consistency in sleep duration over time and an analysis on the association between trajectories of sleep duration between age 50 and 70 and incident multimorbidity (S9 Table). This analysis is now reported and commented as follow in the manuscript:

Results (page 19, lines 348-356):

***“****Third, we examined trajectories of sleep duration. The Pearson correlation of sleep duration at age 50 with sleep duration at age 60 was 0.49 and with sleep duration at age 70 was 0.42 (p<0.001). A moderate correlation was also found between sleep duration at age 60 and age 70 (correlation 0.57, p<0.001). Six trajectories of sleep duration using data on 5510 participants were used and labelled as persistent short sleep, persistent normal sleep, persistent long sleep, change from short to normal sleep, change from normal to long sleep, and change from normal to short sleep (S8 Table). Compared to persistent normal sleep, persistent short sleep duration between age 50 and 70 was associated with increased risk of incident multimorbidity (HR=1.17, 1.01-1.35; p=0.040) in analyses adjusted for sociodemographic, behavioral, and health-related factors (S9 Table).”*

Discussion (page 25, lines 444-446):

*“Using a prospective design, our results show robust evidence of an association of short sleep duration with incident multimorbidity; this was the case for sleep duration measured either in mid or late life or trajectories of sleep duration between age 50 and 70.”*

**5.** Last, what is the correlation between the subjective and objectively measured sleep duration? This should also be feasible to answer with their data and could shed further light into the validity of the findings.

**OUR RESPONSE:** Thank you, we have added this information in the Results section as follows (page 18, lines 322-325):

*“The correlation between accelerometer- and questionnaire-assessed sleep duration was moderate (Pearson correlation=0.41, p<0.001). The shape of the association between accelerometer-assessed sleep duration and incident multimorbidity was similar to that observed with self-reported sleep duration, with the lowest risk of incident multimorbidity seen at 7 hours of sleep (Fig 3).”*

We also comment on this result in the Discussion as follows (page 26, lines 478-482):

*“**First, like most large scale studies on sleep we used self-reported sleep which is likely to be subject to reporting bias. Although the correlation between accelerometer-assessed and self-reported sleep duration was moderate in our study the shape of the association with multimorbidity risk was similar with both measures.”*

**6.** Chronic pain and depressive symptoms are well-known causes of sleeping problems and are, at the same time, closely linked to the development of multimorbidity. In order to untangle these potential confounding effects, could the authors repeat their analyses adjusting for these conditions?

**OUR RESPONSE:** This is an interesting comment and we wish we had the numbers (participants/cases) to examine the hypotheses of chronic pain/depressive symptoms affecting sleep problems that then leads to multimorbidity. This is also a slightly different research question. Furthermore, chronic pain and depressive symptoms are important components of two chronic conditions used to define multimorbidity (depression and arthritis/rheumatoid arthritis, respectively). Adjusting for these conditions would be inappropriate within the current analytic framework. In order to take pre-existing health conditions into account we have added adjustment for the presence of one chronic disease in analyses on sleep duration and incident multimorbidity (Model 3 in the revised manuscript). The associations were slightly attenuated at older ages but remained statistically significant.

Other minor comments:

**7.** In the abstract, the authors mentioned that "Self-reported sleep duration was measured at age 50", but they later clarify this was measured at ages 50, 60 and 70 in the main text. Please update this information in the abstract.

**OUR RESPONSE:** We initially chose to report only results on the association of sleep duration at age 50 and transitions from healthy state, to chronic disease, multimorbidity, and death in the abstract. Given the reviewer’s comment we have revised the abstract to also present findings on the association of sleep duration at age 50, 60, and 70 and risk of incident multimorbidity (page 2, lines 39-43).

**8.** Why do authors call it age 50, 60 and 70 if 10-year approximations have been used? I would have rather worked with (and labelled them as) quinquagenarian, sexagenarian, septuagenarians subjects if the authors wish to make such an approximation.

**OUR RESPONSE:** As described in the Methods section (pages 7-8, lines 133-136), we extracted data so that the measure of sleep duration was from when participants were approximately 50, 60, and 70. In the revised manuscript, we have added mean and standard deviation of the exact age when sleep duration was measured. It was 50.6 (SD=2.6) years for age 50, 60.3 (2.2) for age 60, and 69.2 (1.9) for age 70 (page 12, lines 267, 270 and 272). We feel that using the wording quinquagenarian, sexagenarian, septuagenarians would be misleading as people aged 58 years at sleep measure in the present analysis contribute to the age 60 and not 50. We have used “age 50”, “age 60”, and “age 70” in previous papers using the same approach including one published in Plos Medicine (Gil-Salcedo et al, Plos Med, 2020) and, if the editor agrees, we prefer to keep this wording.

Reference:

Gil-Salcedo A et al. Healthy behaviors at age 50 years and frailty at older ages in a 20-year follow-up of the UK Whitehall II cohort: A longitudinal study. *PLoS Med.* 2020;17(7):e1003147.

**9.** Revise the following sentence in the methods section concerning the dementia outcome: "we used restricted cubic spline regressions with Harrell knots [32], Stata command xblc [33], with 7-hour sleep as the reference to examine the shape of the association between sleep duration and dementia risk".

**OUR RESPONSE:** Apologies, this was a typo, now corrected as multimorbidity risk (page 11, line 233).
