# Supplementary material for: Association of sleep duration at age 50, 60, and 70 years with risk of multimorbidity in the UK: 25-year follow-up of the Whitehall II cohort study
Source: PLoS Med. 2022 Oct 18;19(10):e1004109. doi: 10.1371/journal.pmed.1004109 (PMC9578599; doi:10.1371/journal.pmed.1004109)
Supplement: Attachment — Submitted filename: response-final.docx [file pmed.1004109.s019.docx]

**EDITORS’ COMMENTS**

1. Response to reviewers: Please address the remaining comment of reviewer 4.

**OUR RESPONSE:** We have added a sentence in the limitations section to address the comment of reviewer 4.

**2.** Title: Thank you for revising the title. It may be helpful to also include the study setting in the title: Association of sleep duration at age 50, 60, and 70 with risk of multimorbidity among adults in England: 25-year follow-up of the Whitehall II cohort study”. Or similar.

**OUR RESPONSE:** Thank you, we have revised the title as *“**Association of sleep duration at age 50, 60, and 70 with risk of multimorbidity in the UK: 25-year follow-up of the Whitehall II cohort study”*

**3.** Financial disclosure: Please include the URL of each funder website.

**OUR RESPONSE:** We have added the URL of each funder website as requested.

**4.** Data availability statement: Thank you for revising the data availability statement. If possible, please also include a contact email address where data access questions may be directed.

**OUR RESPONSE:** We have added a contact email address.

**5.** Abstract: Methods and Findings: Please ensure that all numbers presented in the abstract are present and identical to numbers presented in the main manuscript text.

**OUR RESPONSE:** Thank you, we have revised the results section to ensure the numbers presented in the abstract are also presented in the main manuscript (originally some of them were only shown in the tables but not in the text of the main manuscript).

**6.** Abstract: Methods and Findings: Please mention the important covariates adjusted for in the analyses (e.g. socio-demographic measures, behavioral and health-related factors).

**OUR RESPONSE:** This information is provided in line 36 as follows *“Analyses were adjusted for sociodemographic, behavioral, and health-related factors.”*

**7.** Abstract: Methods and Findings: Please report p values to 2 decimal places for values of 0.01 or greater, and to 3 decimal places for values between 0.001 and 0.01.

**OUR RESPONSE:** P values were revised accordingly.

**8.** Abstract: Line 49-50: Please consider mentioning some limitations of the study design, for example the potential for reverse causality, issues with generalizability, or self-reported sleep data could be mentioned here.

**OUR RESPONSE:** We have added the list of limitations as proposed.

**9.** Abstract: Line 54-55: We suggest tempering this sentence slightly, given possibility of reverse causality: “Our findings suggest an association between short sleep duration and multimorbidity.”

**OUR RESPONSE:** Thank you, we have revised the sentence as suggested.

**10.** Author summary: Line 80: We suggest: “There was no clear evidence for an association between long sleep duration at age 50 and risk of chronic disease.”

**OUR RESPONSE:** The sentence has been modified accordingly.

**11.** Lines 514-529: Please remove the Competing interest statement, Funding disclosure statement, and Data availability statement from the main text. Please make sure all information is completely and accurately entered into the manuscript submission system.

**OUR RESPONSE:** This section is now removed from the manuscript and corrected in the online submission system.

**12.** Figure 1: In the legend, please include some explanation for the various transitions (A-E).

**OUR RESPONSE:** Thank you, we have added a legend to Figure 1 with explanations for the various transitions.

**COMMENTS FROM REVIEWER #4**

The authors have adequately responded to my comments.

Regarding my first comment related to the operationalization of multimorbidity based on 13 chronic conditions, while I understand the justification provided by the authors, I still think they should add a limitation in relation to the risk of reverse causality given subclinical health states leading to and departing from a state of multimorbidity. In other words, short sleep duration and sleep disturbances may in fact represent the symptoms of already established disorders (e.g., depression, arthritis) that were still undiagnosed at baseline.

**OUR RESPONSE:** Thank you, we have revised the limitations section to include this point. It now reads:

***“****Fifth,* *despite the use of several covariates residual confounding can be an issue in observational studies. For example, short sleep duration and sleep disturbances may reflect the symptoms of undiagnosed diseases at sleep measures such as depression or arthritis.”*
